# Polymeric nanocapsules loaded with poly(I:C) and resiquimod to reprogram tumor-associated macrophages for the treatment of solid tumors

**DOI:** 10.3389/fimmu.2023.1334800

**Published:** 2024-01-08

**Authors:** Clément Anfray, Carmen Fernández Varela, Aldo Ummarino, Akihiro Maeda, Marina Sironi, Sara Gandoy, Jose Brea, María Isabel Loza, Sergio León, Alfonso Calvo, Juan Correa, Eduardo Fernandez-Megia, María José Alonso, Paola Allavena, José Crecente-Campo, Fernando Torres Andón

**Affiliations:** ^1^ Laboratory of Cellular Immunology, IRCCS Humanitas Research Hospital, Rozzano-Milan, Italy; ^2^ Center for Research in Molecular Medicine and Chronic Diseases (CiMUS), Universidade de Santiago de Compostela, Santiago de Compostela, Spain; ^3^ BioFarma Research Group, CIMUS, Departamento de Farmacología, Farmacia y Tecnología Farmacéutica, Facultad de Farmacia, Universidade de Santiago de Compostela, Santiago de Compostela, Spain; ^4^ Navarra Institute for Health Research (IdiSNA), Program in Solid Tumors, Center for Applied Medical Research (CIMA), Department of Pathology and Histology, University of Navarra, Pamplona, Spain; ^5^ Centro de Investigación Biomédica en Red Cáncer (CIBERONC), Madrid, Spain; ^6^ Centro Singular de Investigación en Química Biolóxica e Materiais Moleculares (CIQUS), Departamento de Química Orgánica, Universidade de Santiago de Compostela, Santiago de Compostela, Spain; ^7^ Instituto de Investigación Biomédica de A Coruña (INIBIC), Oncology Department, Complexo Hospitalario de A Coruña (CHUAC), A Coruña, Spain

**Keywords:** polymeric nanocapsules, poly(I:C), resiquimod (R848), tumor-associated macrophages (TAMs), toll-like receptor (TLR), antitumoral immunotherapy, immunotoxicology, cancer

## Abstract

**Background:**

In the tumor microenvironment (TME), tumor-associated macrophages (TAMs) play a key immunosuppressive role that limits the ability of the immune system to fight cancer. Toll-like receptors (TLRs) ligands, such as poly(I:C) or resiquimod (R848) are able to reprogram TAMs towards M1-like antitumor effector cells. The objective of our work has been to develop and evaluate polymeric nanocapsules (NCs) loaded with poly(I:C)+R848, to improve drug stability and systemic toxicity, and evaluate their targeting and therapeutic activity towards TAMs in the TME of solid tumors.

**Methods:**

NCs were developed by the solvent displacement and layer-by-layer methodologies and characterized by dynamic light scattering and nanoparticle tracking analysis. Hyaluronic acid (HA) was chemically functionalized with mannose for the coating of the NCs to target TAMs. NCs loaded with TLR ligands were evaluated *in vitro* for toxicity and immunostimulatory activity by Alamar Blue, ELISA and flow cytometry, using primary human monocyte-derived macrophages. For *in vivo* experiments, the CMT167 lung cancer model and the MN/MCA1 fibrosarcoma model metastasizing to lungs were used; tumor-infiltrating leukocytes were evaluated by flow cytometry and multispectral immunophenotyping.

**Results:**

We have developed polymeric NCs loaded with poly(I:C)+R848. Among a series of 5 lead prototypes, protamine-NCs were selected based on their physicochemical properties (size, charge, stability) and *in vitro* characterization, showing good biocompatibility on primary macrophages and ability to stimulate their production of T-cell attracting chemokines (CXCL10, CCL5) and to induce M1-like macrophages cytotoxicity towards tumor cells. In mouse tumor models, the intratumoral injection of poly(I:C)+R848-protamine-NCs significantly prevented tumor growth and lung metastasis. In an orthotopic murine lung cancer model, the intravenous administration of poly(I:C)+R848-prot-NCs, coated with an additional layer of HA-mannose to improve TAM-targeting, resulted in good antitumoral efficacy with no apparent systemic toxicity. While no significant alterations were observed in T cell numbers (CD8, CD4 or Treg), TAM-reprogramming in treated mice was confirmed by the relative decrease of interstitial *versus* alveolar macrophages, having higher CD86 expression but lower CD206 and Arg1 expression in the same cells, in treated mice.

**Conclusion:**

Mannose-HA-protamine-NCs loaded with poly(I:C)+R848 successfully reprogram TAMs *in vivo*, and reduce tumor progression and metastasis spread in mouse tumors.

## Introduction

Immunosuppression is a hallmark of cancer with an important impact on tumor progression and therapeutic response (1, 2). The tumor microenvironment (TME) of solid tumors is populated by tumor associated macrophages (TAMs) that limit the ability of the immune system to fight cancer, and their infiltration has been correlated with poor prognosis (3, 4). The protumoral functions of TAMs and their role in the resistance to current antitumoral therapies have been demonstrated (5–7). Macrophages are heterogeneous cells characterized by high phenotypic and functional flexibility (3). As a simplified paradigm, macrophages were originally categorized into two extreme polarizations: M1 and M2 types. TAMs were assigned to an M2-like phenotype, supporting tumor growth, angiogenesis, invasion, metastasis, and resistance to antitumoral therapies (4, 8, 9); or to M1-like macrophages having antitumor activity mediated by their ability to secrete pro-inflammatory cytokines, promote adaptive immune responses and directly kill cancer cells (10–12). Thus, considerable efforts have been put forward to target and modulate TAMs in the TME towards M1-like macrophages with the intention to unleash effective antitumor immune responses.

The pharmacological use of compounds with agonist activity to Toll-Like Receptors (TLR) holds great promise for cancer treatment (13, 14). In the past three decades, only three TLR agonists have been approved by the FDA for cancer treatment: monophosphoryl lipid A (MPL), for papilloma vaccine; imiquimod^®^ (R837), for superficial basal cell carcinoma and the *bacillus* Calmette-Guérin (BCG), for bladder cancer (15). Other agonists of the endosomal TLR3/7/8/9 are under clinical evaluation, and many more in preclinical studies as monotherapy or in combinations (14, 16). Among them, resiquimod^®^ (R848, agonist of TLR7/8) has shown high activity in preclinical settings but failed to prove clinical benefit against genital herpes and hepatitis C (15). In 2011, clinical trials in hematological neoplasia and solid tumors showed controversial results related to poor antitumoral activity and immunotoxic effects, including: fever, fatigue, nausea and cytokine release syndrome (17). To solve these issues, recent efforts have been published using nanotechnological approaches for the controlled release of R848 at the tumor site, preventing systemic immunotoxicity (18–22). Similarly, poly(I:C)-nanoparticle based formulations (i.e. BO-112®) or poly-ICLC (Hiltonol®), a synthetic analogue of dsRNA which mimics RNA from viruses binding to endosomal TLR3, have shown better stability *in vivo versus* the free drug, and are being tested in clinical trials (23–28).

We have recently demonstrated the superior therapeutic efficacy of poly(I:C) combined with R848, *versus* the combination with R837, or any of these TLR agonists as monotherapy, *in vitro* and *in vivo* (29). Our comprehensive analysis of the TME in preclinical lung tumor models demonstrated that the antitumoral activity was mainly driven by macrophage reprogramming towards M1-like antitumor effector cells, which promoted the activation of innate and adaptive immune responses against cancer cells. To advance further, we have considered the loading of both TLR agonists (poly(I:C)+R848) into nanocapsules (NCs) to improve their ability to reach TAMs in the TME, and reduce their side effects. Our group has contributed to this cancer field with the development of polymeric nanocarriers loading cytotoxic drugs, such as docetaxel (30–32), siRNA (33), monoclonal antibodies (34). Also, in the immunotherapy field, we have nanoencapsulated chemokines (35) and poly(I:C) (36). In the vaccine area, we have engineered imiquimod-loaded chitosan NCs (37) and poly(I:C) nanoparticles (NPs) with adjuvant purposes (38, 39). Lastly, aiming to improve delivery towards TAMs in the TME, we have recently reported the synthesis, development and evaluation of hyaluronic acid (HA) NCs functionalized with mannose (Man), achieving high accumulation in solid tumors (40).

Here, we propose the development of a nanocarrier for the *in vivo* administration of the poly(I:C)+R848 combination, delivering both drugs in a single NC formulation towards TAMs in the TME of solid tumors. For this purpose, we initially developed a series of NCs with five different coating polymers loaded with R848 and evaluated their ability to polarize macrophages *in vitro* towards M1-like antitumor effector cells. This screening allowed the selection of the R848-protamine-NCs, with optimal activity, which were then loaded with poly(I:C) and externally coated with an additional layer of hyaluronic acid functionalized with mannose (HA-man) to improve the targeting of TAMs *in vivo*. The protamine NCs loaded with poly(I:C)+R848 were tested in immunocompetent murine models of lung cancer and fibrosarcoma showing satisfactory antitumoral activity and an excellent safety profile.

## Materials and methods

### Materials

D-L-α-tocopherol (Vitamin E) was purchased from EMD Millipore Corp. (CAS#59-02-9, Billerica, MA, USA). D-α-Tocopherol polyethylene glycol 1000 succinate (TPGS) (CAS#9002-96-4) was obtained from Antares Health Products, INC (Jonesborough, TN, USA). Polyarginine (nBu-PArg (150)[HCl], MW 29 kDa, CAS#26982-20-7) was obtained from PTS (Valencia, Spain), protamine sulphate from Yuki Gosei Kogyo (Tokyo, Japan, CAS#9009-65-8), chitosan hydrochloride from Heppe Medical Chitosan GmbH (Halle, Germany, CAS#70694-72-3), polysialic acid (30 kDa) from S.I. India (CAS#7699-41-4) and dextran sulfate from Dextran Products (Toronto, Canada, CAS#9011-18-1). Sodium cholate was acquired from Dextra Laboratories Ltd. (Reading, UK, CAS#206986-87-0), benzethonium chloride was purchased from Spectrum Chemical (New Jersey, USA, CAS#121-54-0) and ethanol from Scharlau (Port Adelaide, SA, Australia, CAS#64-17-5). Resiquimod (R848) was purchased both from Sigma-Aldrich and MedChemExpress and both were used for characterization and cell studies and for *in vivo* only the second one was used. Resiquimod HCl (catalog# tlrl-r848) and HMW poly(I:C) (catalog# tlrl-pic) were acquired from InvivoGen (CA, USA). Ultrapure endotoxin-free water (Milli-Q Integral system from EMD Millipore Corp., Billerica, MA, USA) was used for *in vitro* and *in vivo* studies, while ultrapure water was used for all the other experiments. Phosphate Buffered Saline (PBS) and complete cell culture RPMI media (10% Fetal Bovine Serum - FBS + 1X Penicillin-Streptomycin-Glutamine - PSG) were purchased from Fisher (Massachusetts, USA). Hyaluronic acid was purchased from Lifecore Biomedical (sodium hyaluronate, lot#024168, specification number LDP-9800042; Mw 57 kDa by MALLS). Synthesis of hyaluronic acid functionalized with mannose (HA-man) was performed as recently reported (40).

### Preparation of polymeric nanocapsules

**Figure 1:**
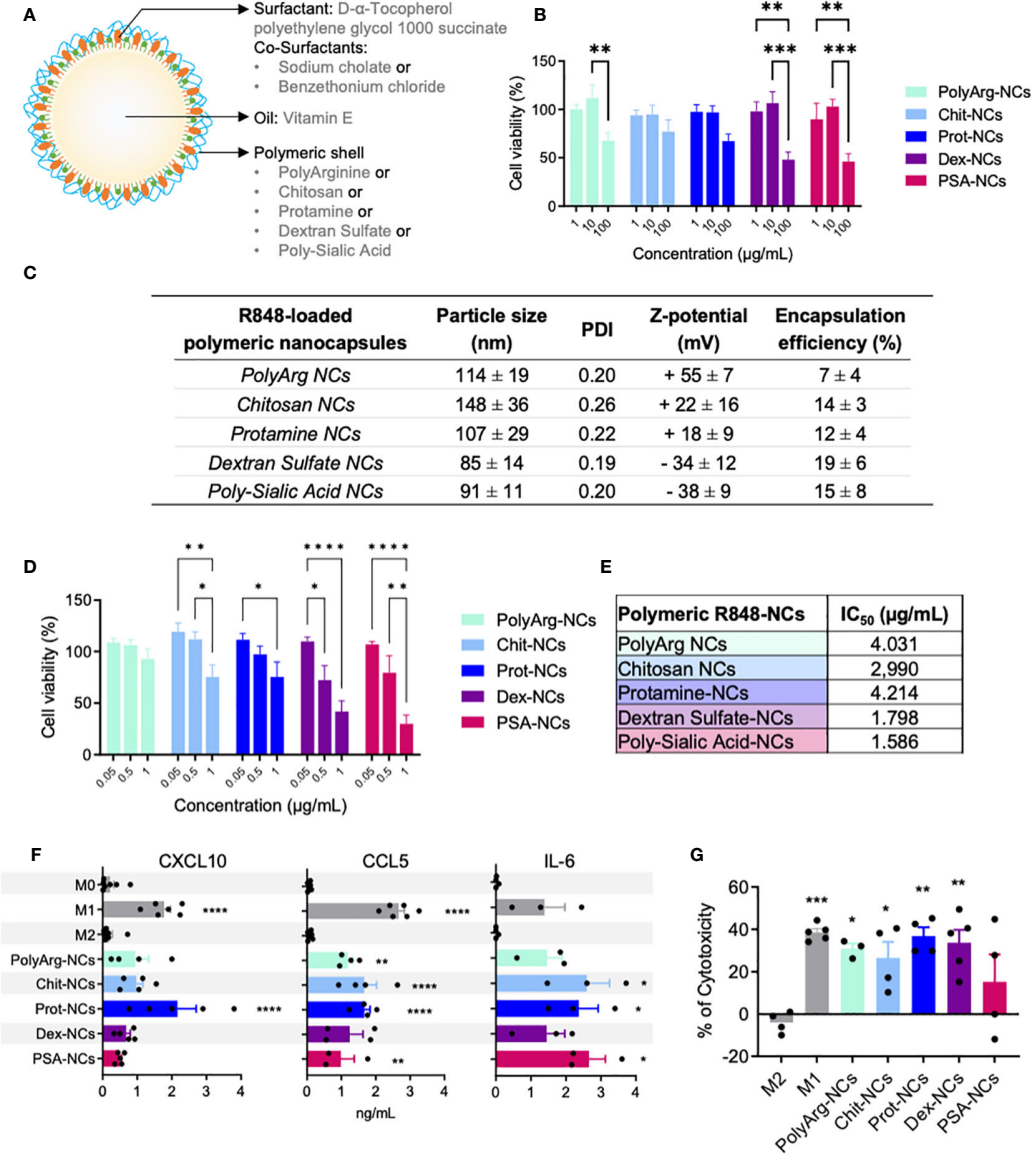
Polymeric nanocapsules: characterization, toxicity and immunomodulatory evaluation *in vitro* using primary human macrophages. **(A)** Schematic representation of the nanocapsules (NCs) and their composition. **(B)** Dose-response toxicity of blank-NCs on HMDMs exposed for 24h (1; 10; 100 µg/mL); N=4. **(C)** Physicochemical characteristics of R848-loaded-NCs prepared with different outer polymers (n ≥ 3). **(D)** Dose-response toxicity on HMDMs exposed for 24h to R848-NCs (0.5 µg/mL); N=9. **(E)** IC50 values of R848-NCs in HMDMs (24h); N=4. **(F)** Secretion of CXCL10, CCL5 and IL-6 by HMDMs exposed for 24h to R848-NCs (0.5 µg/mL). **(G)** Cytotoxic activity of HMDMs, after 48h treatment with R848-NCs (0.5 µg/mL), towards human PANC-1 cells. All values represent mean ± SD. Statistical comparison was performed using a two-way ANOVA followed by a Tuckey’s multiple comparison test. Statistically significant differences are represented as *(p<0.05), **(p<0.01), ***(p<0.001) and ****(p<0.0001). NCs, nanocapsules; PolyArg, polyarginine; Chit, chitosan; Prot, protamine; Dex, dextran sulfate; PSA, poly-sialic-acid.

Polymeric nanocapsules (NCs) were prepared by the solvent displacement method, previously described by our group (41). For the first screening of blank NCs, the organic phase was composed of 27 mg/mL of DL-α-Tocopherol, 8 mg/mL of TPGS, 1 mg/mL co-surfactant (sodium cholate or benzethonium chloride) in ethanol. 500 µL of the ethanolic phase were added over 1.5 mL of the polymer aqueous solution (1.33 mg/mL) in a 5 mL beaker, under magnetic stirring (700 rpm). The final composition of the blank-NCs is shown in **Figure 1A** and **Table S1**. To reduce the particle size and benefit the *in vivo* biodistribution, the solvent displacement method was slightly modified by injecting the organic phase into the aqueous phase. Using this method, R848 was loaded into protamine NCs. For this, the following solutions were prepared: a) ethanolic solution of vitamin E (135 mg/mL), TPGS (40 mg/mL) and sodium cholate (10 mg/mL), b) ethanolic solution of R848 (12 mg/mL). 100 µL of each of these solutions were mixed and added over 1.8 mL of an aqueous solution of protamine (1.11 mg/mL). The organic phase was injected into the aqueous phase with a 0.2 mL syringe myjector U-100 insulin 0,5 mL 29G x ½” – 0.33x12mm from Terumo (Elkton, USA), under magnetic stirring (900 rpm). The stirring was maintained for 10 minutes and then the solvent was removed by evaporation at room temperature into the fume hood. Volume was readjusted to the initial 2 mL with ultrapure water in a volumetric flask. After this, NCs were isolated and concentrated by ultrafiltration using Amicon filtration device (cut-off 100 kDa) purchased from Merck (Darmstadt, Germany). The centrifugation was performed at 7000 g and 15 °C. Samples were washed twice by adding 1 mL of water and re-centrifuging under the same conditions. The isolated NCs were taken to a final concentration of R848 of 0.25 mg/mL with ultrapure water (after quantifying the concentration of R848 by HPLC, see below “encapsulation efficiency”). For the association of poly(I:C), the NCs were diluted until a R848 concentration of 0.3 mg/mL. The poly(I:C) association to R848-loaded protamine NCs was implemented by simple incubation, adding 300 µL of 0.3 mg/mL poly(I:C) aqueous solution to 300 µL of protamine NCs (0.3 mg/mL of R848), with a final concentration of 0.15 mg/mL for both drugs. NCs were then concentrated again by ultrafiltration with the Amicon filtration device (cut-off 100 kDa) purchased from Merck (Darmstadt, Germany) until a 0.3 mg/mL concentration of both drugs was reached. For *in vivo* experiments, NCs were diluted with endotoxin-free water until a final concentration of 0.25 mg/mL for both drugs. In addition, the NCs were used at a concentration of 0.3 mg/mL for their coating with HA or HA-mannose. A solution of HA or HA-mannose (5.4 mg/mL) was prepared in water. 0.6 mL of this solution was added over 3 mL of poly(I:C)+R848-NCs in a glass vial under magnetic stirring and let stir for 30 minutes, reaching the final concentration for both drugs of 0.25 mg/mL.

### Characterization of polymeric nanocapsules

Particle size and PDI were measured by Dynamic Light Scattering (DLS), and zeta potential values by Laser Doppler Anemometry (LDA), both using a Zetasizer Nano ZS de Malvern Instruments (Malvern, UK). Asymmetrical flow field-flow fractionation (AF4) was used to confirm particle size and dispersion. Concentrated samples were diluted 1:50 with ultrapure water prior to their characterization. The Nanosight NS300 System (Malvern, UK) was used to measure the size and concentration of NCs, using the Nanoparticle Tracking Analysis (NTA) version 3.3 Dev Build 3.3.104 with a sCMOS camera and a Blue488 laser. Samples were diluted in water (1:10000) and pumped with a syringe at speed of 40. The movement of the particles in the sample was recorded 5 times for 1 minute each time at 25°C.

### Evaluation of encapsulation efficiency of R848 by HPLC and poly(I:C) by electrophoresis

Encapsulation efficiency was directly measured in the isolated samples by HPLC. Prior to quantification, NCs were treated with acetonitrile in a 1/31 v/v ratio (NCs : ACN), for 10 min under orbital stirring, to destroy them and extract the R848. For the quantification, samples were diluted with the mobile phase (1:5 sample/mobile phase v/v) and analyzed by HPLC (Hitachi, ELITE LaChrom), using an ACE Equivalence 5 µm C18 column at 30°C. Samples were run with an isocratic regimen (1.5 mL/min) of 70% acetate buffer pH 3.7 and 30% of ACN. R848 retention time was 3.5 min. Detection was performed with UV detector at 239 nm. Encapsulation efficiency was calculated as:


$$EE\%=\frac{Encapsulated\;R848}{Total\;theoretical\;R848}x100$$

Poly(I:C) was qualitatively determined in agarose gel at 1% w/v in Tris Acetate-EDTA buffer, with or and without the incubation, using an excess of anionic competitor (heparin) for poly(I:C) displacement. Each lane was loaded with 2.03 µg of poly(I:C) and with 1xSYBR^®^Gold nucleic acid stain. For the displacement with heparin, NCs were incubated for 30 min at 37°C in a 64:1 w/w ratio heparin to poly(I:C). As control lanes, a DNA 1kb ladder and free poly(I:C) in the same conditions were added. Gels were run for 45 min at 90 V in a Sub-Cell GT cell 96/19, evaluated with an UV transilluminator and analyzed with Image Lab™ Software. Same protocol was performed for release studies, adjusting the volumes to 2.04 µg of poly(I:C).

### Stability of polymeric nanocapsules

Colloidal stability of the NCs was tested by measuring the particle size and PDI in relevant conditions. The samples were tested in suspension at room temperature in PBS with 10% FBS diluting the samples 1:50 or 1:10, in complete cell culture media, or in storage conditions in suspension keeping the samples at 4°C. Particle size and PDI were measured at selected time points (hours or weeks).

### Cell culture models: human primary macrophages and cancer cell lines

Primary human monocyte derived macrophages (HMDMs) were prepared as previously described (42). Briefly, monocytes were isolated from the blood of healthy donors through density gradients. M0 macrophages were obtained by culturing 1×106 cells/mL human monocytes for 5 days in 5% FBS/RPMI supplemented with 25 ng/mL of recombinant human M-CSF (rhM-CSF; Cat#300-25, PeproTech, London, UK). M1 macrophages were polarized by stimulating M0 macrophages with LPS (100 ng/mL) (Sigma, Cat#L4005) and IFN-γ (50 ng/mL) (Cat#300-02, PeproTech, London, UK) for 24 h, and M2 macrophages were obtained by polarizing M0 macrophages with IL-4 (20 ng/mL) (Cat# 200-04, PeproTech, London, UK) for 24 h. Murine lung adenocarcinoma CMT167 (ECACC), human pancreatic carcinoma PANC1 (ATCC), murine fibrosarcoma MN/MCA1 cells and human monocytic THP1 cell lines (ATCC) were cultured in DMEM (Lonza) supplemented with 10% fetal bovine serum (Eurobio), 2 mM glutamine and 100 U/mL penicillin/streptomycin (Sigma-Aldrich). THP1 cells were treated for 24 hours with 30 nM phorbol myristate acetate (PMA, InvivoGen, Cat#tlrl-pma) for their differentiation as a model of M0 macrophages (43, 44). For M1 and M2 polarization THP1 cells were treated with LPS+IFN-γ (100 ng/mL and 50 ng/mL) or IL-4 (20 ng/mL), respectively. At 80-90% confluence, cells were washed with PBS, detached with trypsin (Sigma-Aldrich) and then distributed into new culture flasks with fresh medium and cultured at 37°C and 5% CO_2_.

### Cell viability/toxicity studies *in vitro*

Macrophages (105 cells/well), THP1 (0.9x105 cells/well) or CMT167 cells (0.2x105 cells/well) were cultured in 96 well black microplates (Corning, Cat#3875) and treated in appropriate cell culture medium. At the indicated times, cell viability was determined by Alamar Blue assay (Invitrogen, Cat#DAL1100), following manufacturer’s protocol. Fluorescence intensity at λ_abs_ 560 nm and λ_em_ 590 nm was measured with a Synergy H4 plate reader (BioTek). Values were normalized to non-treated cells used as controls and considered as 100% cell viability. Cell viability was calculated according to the following equation:


Cell viability (%)=(1− fluorescence / control fluorescence)×100

For the calculation of IC_50_ (half maximal inhibitory concentration at which 50% of cells are dead), the toxicity was measured by Alamar Blue assay after treatment with concentrations of 0, 0.1, 1, 10, 100 and 1000 *μ*g/mL of total NC-components, while concentrations of 0.05, 0.5, 1, 5 and 10 *μ*g/mL of R848 were tested for the loaded-NCs. Correspondence between blank and R848-loaded-NCs is presented in **Table S2**.

### Quantification of cytokine secretion

Supernatants from primary human macrophages were collected after 24 hours exposure to indicated treatments. Cytokine secretion (human CXCL10, CCL5, IL-6) was measured by commercially available ELISA kits according to manufacturer’s instructions (R&D Systems, Cat#DY266, DY278, DY206). Plasma samples from mice were obtained at the end of *in vivo* experiments, and levels of murine IL-6, CXCL10 and TNF-α were quantified using a customized MILLIPLEX MAP kit (Merck-Millipore, Cat# HCYTOMAG-60K) according to the manufacturer´s instructions and run on a Luminex MAGPIX machine (Merck-Millipore).

### Cytotoxic activity of primary human macrophages towards PANC-1 human pancreatic cancer cells

The cytotoxicity of pre-treated macrophages towards cancer cells was performed as recently described (40). Briefly, primary human monocyte derived macrophages were seeded in 24 well plates (106/well) and exposed to indicated treatments (5 μg/mL of poly(I:C) and/or R848 loaded into protamine-NCs) for 24 hours. For the controls, macrophages were treated with LPS/IFN-γ or IL-4 to polarize them towards M1 or M2 phenotypes. Macrophages were then washed and co-incubated for 2 days with tumor PANC-1 cells (2.5x104), previously stained with CellTraceTM Far Red 1 mM (Invitrogen, Cat#C34564). Cells were then trypsinized, collected and fixed in 1% PFA-PBS for their flow cytometry analysis using a FACS Canto II Instrument (BD Biosciences). For the flow cytometry analysis, acquisition was set to 45 seconds and the number of high fluorescence intensity events (corresponding only to proliferating tumor cells) were counted for each sample and normalized to the non-treated (M0) macrophages. The percentage of cytotoxicity is presented according to the following equation:


%of cytotoxicity = 100-(N° of cancer cells / N° of cancer cells in control)×100

### Cytotoxic activity of murine splenocytes towards CMT167 murine lung cancer cells *ex vivo*

Splenocytes of tumor-bearing mice were immediately collected after sacrifice, processed and disaggregated following an enzymatic/mechanic procedure: cut into small pieces, digested with 0.5 mg/ml Collagenase IV from *Clostridium histolyticum* (Sigma, Cat# C5138) in RPMI without serum for 10 minutes at 37°C in an orbital shaker incubator; cell suspension was passed through a 70-μm cell strainer to remove any undigested tissue. Cells were incubated in ACK lysis buffer (Lonza, Cat#10-548e) for 5 min at room temperature to remove red blood cells; then centrifuged at 4°C, 300 rcf for 5 minutes and the pellet was resuspended in complete RPMI. Splenocytes were cultured in 24 well plates (106/well), treated *ex vivo* for 24 hours, then incubated for 48 hours with stained CMT167 murine lung cancer cells (2x104) and analyzed by FACS, as described in the previous section.

### Animals and *in vivo* experiments

Experiments involving animals were conducted following recently published protocols (45), in accordance to national (4D.L. N.116, G.U. 1992) and international law and policies (EEC Council Directive 2010/63/EU, OJL 276/33, 22-09-2010; NIH Guide for the Care and Use of Laboratory Animals, US National Research Council, 2011). Authorization was obtained from the Italian Ministry of Health number 453/2020-PR (prot. 6B2B3.103). Six-week-old female C57BL/6 mice were purchased from Charles River Laboratories and maintained in specific-pathogen-free (SPF) facility. To generate the lung cancer models, 10^5^ CMT167 cells were implanted in the right flank of C57BL/6 mice for the subcutaneous model or intravenously for the orthotopic lung cancer model. To generate the orthotopic fibrosarcoma model, 105 MN/MCA1 cells were injected into the caudal thigh muscle. For each treatment group, 5-7 animals were used. Tumor volume was measured with a digital caliper using the following formula: *tumor volume ≈ (width^2^×length/2).* The NCs were administered by intratumoral injection (100 μL) or intravenous (100 μL) injection, as indicated for each experiment. Survival was monitored daily, and tumor volume was measured until maximum allowed size or at the end of the protocol. At sacrifice, primary tumors, lungs and spleens were excised and weighted. Surface lung macrometastasis were visually counted on lungs fixed in Bouin’s solution. Relevant organs were stained by hematoxylin and eosin for *in vivo* histopathological evaluation of tissue toxicity.

### Flow cytometry

On sacrifice of mice, tumors were prepared for FACS analysis as described in ref (45). Cells were stained with LIVE/DEAD Fixable Aqua Dead Cell Stain (Invitrogen, 1:1000 in PBS −/−) for 30 min at room temperature (RT). Cells were then stained with the antibodies mix, provided in online **Supplementary Table S3**, in FACS buffer for 30 min at 4°C. Cells were washed with FACS buffer and fixed with FACS FIX Buffer (1% PFA PBS) for 20 min at 4°C until analysis. Cells were analyzed on FACS Canto II and LSR Fortessa, and data were generated by FACS Diva software (BD Biosciences).

### Multiplexed tumor immunophenotyping

For multispectral immunophenotyping, the murine-specific kit from Akoya (NEL840001KT) was used as previously described (45), with additional markers that were evaluated in two separate panels. In the first panel, the following primary antibodies were used: anti-FOXP3, anti-CD4 and anti-CD8; the second panel included: anti-F4/80, anti-CD86 and anti-Arginase-1. The source and dilution of the antibodies as well as other experimental details can be found in **Supplementary Table S4**. Sample scanning, spectral unmixing and quantification of signals were conducted with the Vectra Polaris Automated Quantitative Pathology Imaging System (Akoya), using the Phenochart (Akoya) and QuPath-0.4.3 software. Data were given as number of cells with a specific immunophenotype/total number of cells.

### Statistical analysis

Data analysis was performed using GraphPad Prism V.8 (GraphPad Software). Description of statistical comparisons are provided in each figure’s legend.

## Results

### Design, preparation, characterization, and *in vitro* evaluation of a panel of polymeric nanocapsules

A panel of polymeric nanocapsules (NCs) with different outer polymers was prepared and their interaction with macrophages (HMDMs and THP1) was evaluated *in vitro*. Five natural polymers with favorable biocompatible and biodegradable profile were selected, including: two polypeptides (polyarginine and protamine) and three polysaccharides (chitosan, polysialic acid and dextran sulphate). The solvent displacement technique was implemented to prepare the NCs (41). Their basic structure is depicted in **Figure 1A** and better detailed in **Table S1**. These blank-NCs showed a particle size around 150 nm for all the prototypes, with low polydispersity index (PDI < 0.2) and a surface charge determined by the net charge of the outer polymer (**Figure S1A**).

M0/M1-like/M2-like macrophages were exposed to the NCs for 24 hours or just 1 hour prior to assay their viability using Alamar Blue (**Tables S5, S6**). These dose-response experiments showed no significant toxicity for the chitosan and protamine NCs up to 100 µg/mL, while the other prototypes (polyarginine, dextran sulfate and polysialic acid NCs) presented significant toxicity at the same dose towards M0 macrophages (**Figure 1B**). In a kinetics experiment (1, 24 and 48 hours) using 10 µg/mL of blank-NCs, the same M0 cells showed no changes up to 24 hours, while at 48 hours significant toxicity was observed only for the polyarginine-NCs (**Figure S1B**). **Tables S2** and **S3** demonstrate the expected higher sensitivity of primary human monocyte derived macrophages (HMDMs) when compared to the THP1 cell line. Of note, polyarginine and polysialic acid NCs, with the highest surface charge (+50) and the lowest one (-42), respectively, showed the highest toxicity (lowest IC_50_). As a whole, it seems very clear that protamine-NCs are non-toxic (IC50 > 1000 µg/mL) and show the best biocompatibility with macrophages *in vitro* (**Figures 1B**, **S1B, S1C, Tables S2, S3**).

### Preparation and screening *in vitro* of polymeric nanocapsules loaded with R848

Before the loading of the drug (R848) into the NCs, we further tried to reduce their particle size, looking for an improved biodistribution and tumor accumulation *in vivo* (46–48). A particle size below 100 nm was achieved for the all the blank-NC-prototypes (**Figure S2**), only with the slight modification of the solvent displacement method by changing the addition of the ethanolic phase over the aqueous phase, from pouring (low addition rate) to injecting (high addition rate) and maintaining constant the initial concentrations of each component (49, 50). All blank-NCs were stable in complete cell culture media at 37°C and in storage conditions at 4°C (**Figure S3**).

After optimization of the particle size, R848 was loaded into the oily core of the NCs. Due to its lipophilic nature, the drug was incorporated into the organic phase and the NCs were prepared by the modification of the solvent displacement method (high addition rate), maintaining a particle size around 100 nm. **Figure 1C** shows the physicochemical characterization of the R848-loaded prototypes after the purification process of ultrafiltration. While chitosan-NCs maintained their higher particle size (150 nm) the other prototypes presented smaller size (closer to 100 nm). The R848 encapsulation efficiency (EE) was evaluated after NC-solubilization in ethanol by HPLC, showing values between 10 and 20%, except for the polyarginine-NCs with the lowest loading (7 ± 4%). Differences in the EE of these prototypes might be explained by the influence of the polymer shell in the pH and the different surfactants which affect drug solubility. In cell culture media, all R848-loaded-NCs were stable for 3 days, except chitosan-NCs, showing increase in size up to 200 nm at 24 hours (**Figure S4A**). At 4 °C (storage conditions), all the prototypes showed no changes in particle size up to 6 months (**Figure S4B**).

All NC preparations were routinely tested for endotoxin contamination using the LAL assay; sporadic contaminated NCs were discarded and not used in the experiments *in vitro* or *in vivo. In vitro* toxicity testing of R848-loaded-NCs was performed at 24 hours using HMDMs (**Figure 1D**), THP1 (**Figure S6A**) and CMT167 lung cancer cell lines (**Figure S6B**) by Alamar Blue assay. No significant toxicity was found for any prototype towards THP-1 or CMT167 cells, while in the case of HMDMs all NCs showed lower cell viability at 24 hours with the highest dose of R848 (1 µg/mL). Curiously, at this dose (1 µg/mL) the polyarginine-NCs did not show toxicity towards HMDMs (**Figure 1D**) but presented significant toxicity towards the THP-1 cell line (**Figure S6A**). The NCs with negative surface charge (dextran sulfate and polysialic acid NCs) (**Figures 1D, E**) were more toxic, while the protamine-NCs presented the lower toxicity (higher IC_50_). R848-NCs with positive surface charge were non-toxic towards HMDMs at 24 hours up to 0.5 µg/mL, equivalent to approximately 30 µg/mL of total components of NCs (see **Table S2** for NC concentrations). Therefore, this dose was used in the subsequent experiments *in vitro*.

Next, we tested the ability of the R848-loaded-NCs to activate the NF-κB pathway using a THP-1-NF-κB reporter cell line. After exposure for 24 hours to R848-loaded-NCs, dose-response experiments demonstrated the higher NF-κB activation for the protamine, dextran and polysialic acid NCs, in this order, with no significant activity for the chitosan or polyarginine NCs (**Figure S7**). To further assess the polarization of treated macrophages, we evaluated the secretion of pro-inflammatory cytokines (M1 markers) from HMDMs in response to the R848-loaded-NCs (**Figure 1F**). All types of R848-loaded-NCs successfully stimulated macrophages to produce the T cell attracting chemokines CXCL10 and CCL5, and IL-6, with the protamine-R848-NCs having the best efficacy to induce CXCL10 secretion. Finally, we performed functional assays to evaluate the ability of the R848-loaded-NCs to polarize HMDMs towards a pro-inflammatory M1 anti-tumoral phenotype that kills cancer cells. M0 macrophages were exposed to the NCs for 24 hours and then co-cultured with tumor cells for 48 hours; the residual number of alive cells was quantified by FACS. As controls, classical M1 macrophages expressed significant cytotoxic activity towards cancer cells while M2 macrophages did not. Macrophages treated with polyarginine, chitosan, protamine and dextran sulfate NCs enhanced the cytotoxic activity of macrophages to a level comparable to M1-antitumoral macrophages (**Figure 1G**). As a whole, these results suggest that, considering the balance between toxicity and functional polarization *in vitro* of human macrophages, protamine-R848-NCs are the most promising candidates for the treatment of cancer *in vivo*.

### Dual loading of the TLR agonists poly(I:C)+R848 in protamine nanocapsules: preparation, characterization and *in vitro* activity

**Figure 2:**
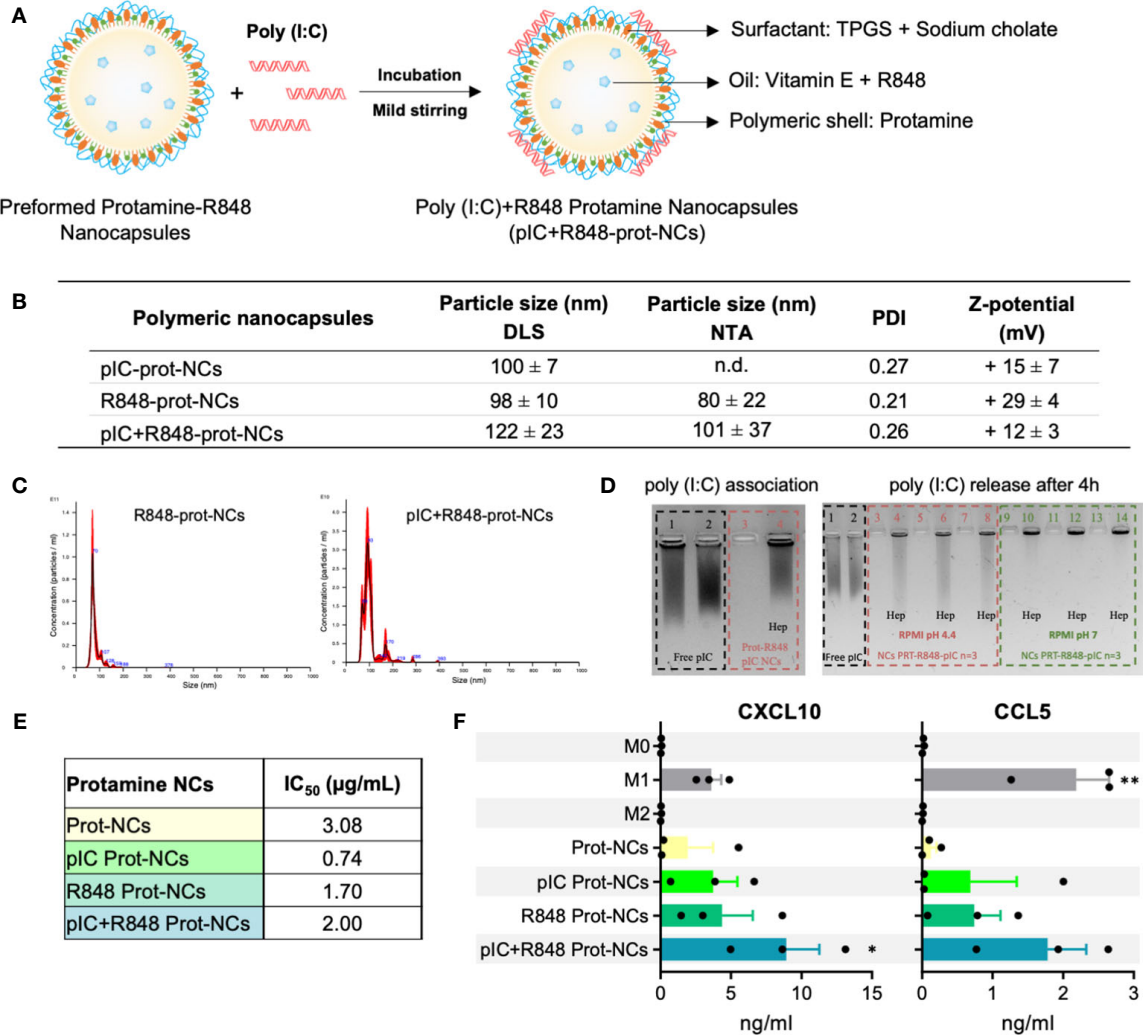
Synthesis and characterization of poly(I:C)+R848-loaded-protamine-nanocapsules. **(A)** Schematic representation for the addition of poly(I:C) on the surface protamine coating of the R848-NCs, and description of their components. **(B)** Physicochemical characterization of protamine-NCs loaded with poly(I:C) and/or R848, and coated with HA or HA-mannose (n ≥ 3). **(C)** Asymmetrical flow field-flow fractionation (AF4) comparison of R848-loaded protamine nanocapsules (R848-prot-NCs) (left) and poly(I:C)+R848-loaded protamine nanocapsules (pIC+R848-prot-NCs) (right). **(D)** (left gel) Agarose gel retardation assay to evaluate poly(I:C) binding to the R848-prot-NCs. Controls: (1) free poly(I:C) and (2) free poly(I:C) with heparin; (3) pIC+R848-prot-NCs and (4) pIC+R848-prot-NCs with heparin. (right gel) Agarose gel retardation assay to evaluate the release and integrity of poly(I:C) after 4 h of incubation in cell culture media at 37°C at acid and neutral pH (dilution ½). Controls: (1) free poly(I:C) and (2) free poly(I:C) with heparin; (3, 5, 7) pIC+R848-prot-NCs in RPMI pH 4.4 and (4, 6, 8) with heparin, (9, 11, 13) pIC+R848-prot-NCs in RPMI pH 7 and (10, 12, 14) with heparin. **(E)** IC50 values of poly(I:C) and/or R848-loaded-prot-NCs in HMDMs for 24 hours. **(F)** Secretion of CXCL10 and CCL5 by HMDMs exposed 24 hours to poly(I:C) and/or R848-prot-NCs (0.5 µg/mL). All values represent mean ± SD. Statistical comparison was performed using a two-way ANOVA followed by a Tuckey’s multiple comparison test. Statistically significant differences are represented as *(p<0.05) and **(p<0.01). pIC, poly(I:C); R848, resiquimod; prot-NCs, protamine nanocapsules.

Our next step was the combination of R848 with poly(I:C) in the same nanocarrier, looking for the synergistic effect of the TLR3 + TLR7/8 agonists and thus increase the therapeutic activity (29). The incorporation of the negatively charged poly(I:C) was performed by simple incubation and its adsorption on the surface of the positively charged protamine-R848-NCs (**Figure 2A**). Protamine is an arginine-rich polypeptide with positive charge commonly used to nanocomplex different types of nucleic acids with cell penetration properties (51), while poly(I:C) is a dsRNA with negative charge, thus their association by electrostatic interaction is feasible. A solution of poly(I:C) was incubated with pre-formed R848-loaded-protamine-NCs, leading to the adsorption of the anionic dsRNA onto the surface of the positive NCs (**Figure 2A**). To establish the optimal ratio dsRNA:protamine, different initial concentrations of dsRNA were added to blank NCs, with theoretical charge ratios from 1:5 to 1:25 (data not shown). All conditions led to the formation of NCs with particle size around 100 nm, with the exception of the highest concentration of poly(I:C) that led to aggregation. Thus, a charge ratio poly(I:C):protamine 1:14 was selected, showing an effective adsorption of poly(I:C) without excessive dilution of R848. These NCs showed a particle size of 122 ± 23 nm, with a reduction in the zeta potential from +29 ± 4 mV to + 12 ± 3 mV, and low polydispersity index (PDI <0.3) (**Figure 2B**). Their characterization was further studied by other techniques, such as NTA and AF4, confirming the particle size and monodisperse population (**Figures 2B, C**). Poly(I:C) association on the surface of the NCs was indicated by the change in surface charge and confirmed by electrophoresis (**Figure 2D**). The polyacrylamide gel showed no free poly(I:C), which was effectively released after the incubation of NCs with anionic heparin, that competes with the poly(I:C) for the positive charges of protamine. These results demonstrate a poly(I:C) encapsulation efficiency close to 100% and higher than the reported for PLGA-based nanocarriers (52). Incubation of the NCs in cell culture media at different pH levels showed no release of poly(I:C) after 4 hours at pH=7, while some release was found at pH=4.4 (**Figure 2D**). Of note, this could be beneficial for the release of the drug in the acidic tumor microenvironment (53), but also at the intracellular level inside acidic lysosomes where the TLR3 receptor is located (36).

We tested the poly(I:C)+R848-protamine-NCs in terms of toxicity and ability to polarize macrophages towards M1-like antitumor effector cells. Using HMDMs, IC50 was determined as 2 µg/mL for poly(I:C)+R848-prot-NCs (**Figure 2E**). Surprisingly, the protamine-NCs loaded with both drugs showed lower toxicity than poly(I:C)-prot-NCs (IC50 = 0.74) or R848-prot-NCs (IC50 = 1.70) furtherly encouraging their safe use for reprogramming TAMs. Moreover, secretion of CXCL10 and CCL5 by HMDMs treated with poly(I:C) and/or R848 loaded protamine NCs was significantly higher with both drugs co-loaded into the same nanocarrier (**Figure 2F**), *versus* protamine-NCs loaded with one drug, confirming the better performance in terms of macrophage M1-polarization for the poly(I:C)+R848-prot-NCs. Thus, we decided to test these prototypes *in vivo* by intratumoral injection as described below.

### Intratumoral administration of poly(I:C)+R848-protamine-nanocapsules: antitumoral efficacy *in vivo*, on the primary tumor and lung metastasis

**Figure 3:**
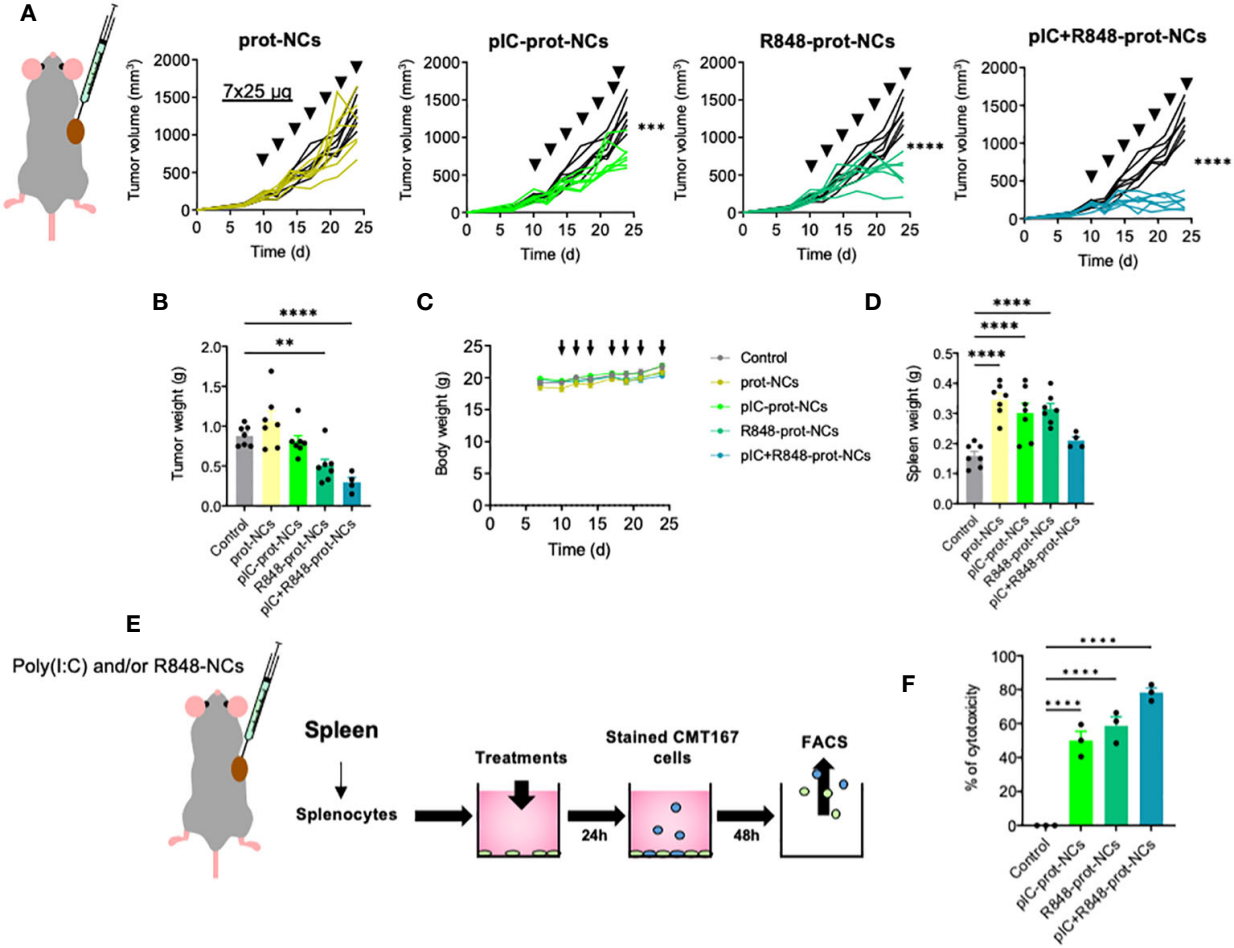
Antitumoral efficacy of poly(I:C) and/or R848 protamine-loaded-NCs using the lung cancer murine model CMT167. **(A)** Evolution of primary tumor growth in CMT167 tumor-bearing mice (s.c.) treated with the blank or drug-loaded-prot-NCs (7 intratumoral injections corresponding to 25 µg of each drug at indicated times). **(B)** Comparison of the tumors weight at sacrifice. **(C)** Weight of whole animals along the experiment, measured 3 times per week **(D)** Comparison of spleen weight of mice at sacrifice. **(E)** *Ex vivo* re-stimulated splenocytes: cytotoxicity towards lung cancer cells (CMT167). Schematic representation of the experimental protocol. **(F)** Cytotoxic activity of splenocytes towards CMT167 cells. Values represent mean ± s.e.m. Statistical comparison was performed using a one-way ANOVA followed by a Tukey’s multiple comparison test. Statistically significant differences are represented as **(p<0.01) and ****(p<0.0001). pIC, poly(I:C); R848, resiquimod; prot-NCs, protamine nanocapsules.

We tested the efficacy of the NCs to prevent tumor progression using an *in vivo* model of CMT167 murine lung cancer cells subcutaneously implanted in C57BL/6 immunocompetent mice (**Figure 3A**). The intratumoral (i.t.) injection of poly(I:C)+R848-prot-NCs, showed potent reduction in tumor volume and weight compared with control mice, while the poly(I:C)-prot-NCs or the R848-prot-NCs (loaded with only one TLR agonist) presented much lower antitumoral activity (**Figures 3A, B**). No effects in terms of tumor growth were observed for the blank-prot-NCs. Along the whole experiment, we monitored the behavior and weight of mice, showing no alterations and thus, no apparent toxicity for any experimental group (**Figure 3C**). However, we found significant changes in the spleen of mice treated with some of the NCs *versus* the control (receiving i.t. injections of PBS). Surprisingly, spleen weight presented around 2-fold increase in mice treated with blank-prot-NCs, poly(I:C)-prot-NCs or R848-prot-NCs, but not in the mice treated with the poly(I:C)+R848-prot-NCs (similar to control) (**Figure 3D**). From these mice, the splenocytes were isolated to investigate their possible activation. Splenocytes include a variety of immune cells (macrophages, dendritic cells, NK, T and B cells) which can be polarized towards an antitumoral or protumoral mode (tolerogenic splenic niche driven by the tumor) (54). Comparing splenocytes from control mice *versus* the same cells isolated from treated mice and further exposed *ex vivo* to the same treatment (as described in **Figure 3E**), the highest antitumoral activity towards lung CMT167 cancer cells, *in vitro*, was clearly observed for the splenocytes isolated from mice treated with poly(I:C)+R848-prot-NCs (**Figure 3F**).

**Figure 4:**
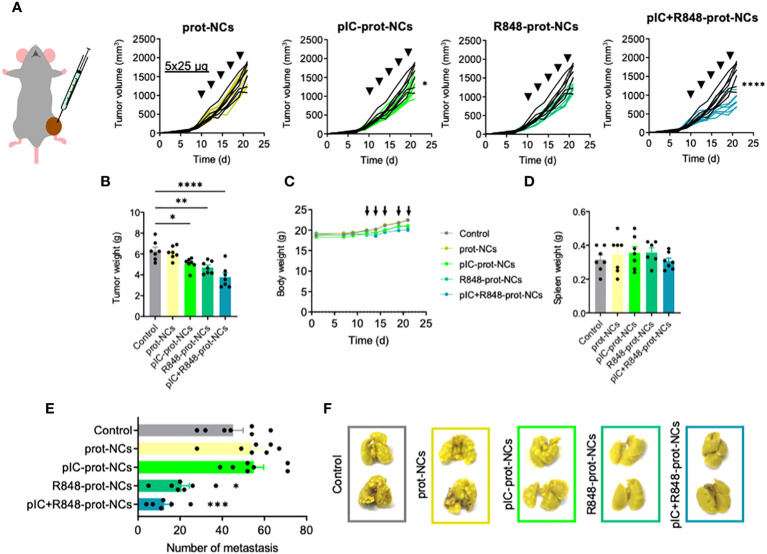
Antitumoral efficacy of poly(I:C) and/or R848 protamine-loaded-NCs on primary tumor and lung metastasis, using the fibrosarcoma murine model MN/MCA1. **(A)** Evolution of primary tumor growth in MN/MCA orthotopic fibrosarcoma-bearing mice treated with the blank or drug-loaded-prot-NCs (5 intratumoral injections corresponding to 25 µg of each drug at indicated times). **(B)** Comparison of the tumors weight at sacrifice. **(C)** Weight of whole animals along the experiment, measured 3 times each week **(D)** Comparison of spleen weight of mice at sacrifice. **(E)** Number of surface lung macrometastasis at sacrifice. **(F)** Representative pictures of two lungs from each treatment group. Values represent mean ± s.e.m. Statistical comparison was performed using a one-way ANOVA followed by a Tukey’s multiple comparison test. Statistically significant differences are represented as *(p<0.05), **(p<0.01), ***(p<0.001) and ****(p<0.0001). pIC, poly(I:C); R848, resiquimod; prot-NCs, protamine nanocapsules.

For a better understanding of local and systemic antitumoral efficacy of NCs loaded with TLR ligands, we used the orthotopic murine fibrosarcoma MN/MCA1 model, a fast-growing and aggressive tumor spontaneously metastasizing to the lungs (55). MN/MCA1 cells were orthotopically (intramuscularly) implanted and mice were i.t. injected with poly(I:C)+R848-prot-NCs. Although tumor growth inhibition was less marked than in the CMT167 lung cancer model, poly(I:C)+R848-prot-NCs still presented a significant antitumoral effect (**Figures 4A, B**). Minor antitumoral activity was found for the poly(I:C)-prot-NCs, but not for the R848-prot-NCs at the same doses, in terms of tumor weight (**Figure 4B**). No apparent toxicity was observed for any group of treatment, in terms of mouse weight or spleen weight (**Figures 4C, D**) and no alterations were observed in mouse behavior. At sacrifice, we collected the lungs and evaluated the number of surface lung macrometastasis in the fibrosarcoma-bearing mice, showing strong metastasis reduction for the poly(I:C)+R848-prot-NCs, and also for the R848-prot-NCs, but not for the poly(I:C)-prot-NCs (**Figures 4E, F**). As a whole, these results demonstrate in two different murine tumor models that intratumoral injection of prot-NCs loaded with both poly(I:C)+R848 present the best antitumoral and antimetastatic activity.

### Intravenous administration of protamine nanocapsules loaded with poly(I:C)+R848 and coated with HA-man: antitumoral efficacy *in vivo* and reprogramming of the tumor microenvironment

**Figure 5:**
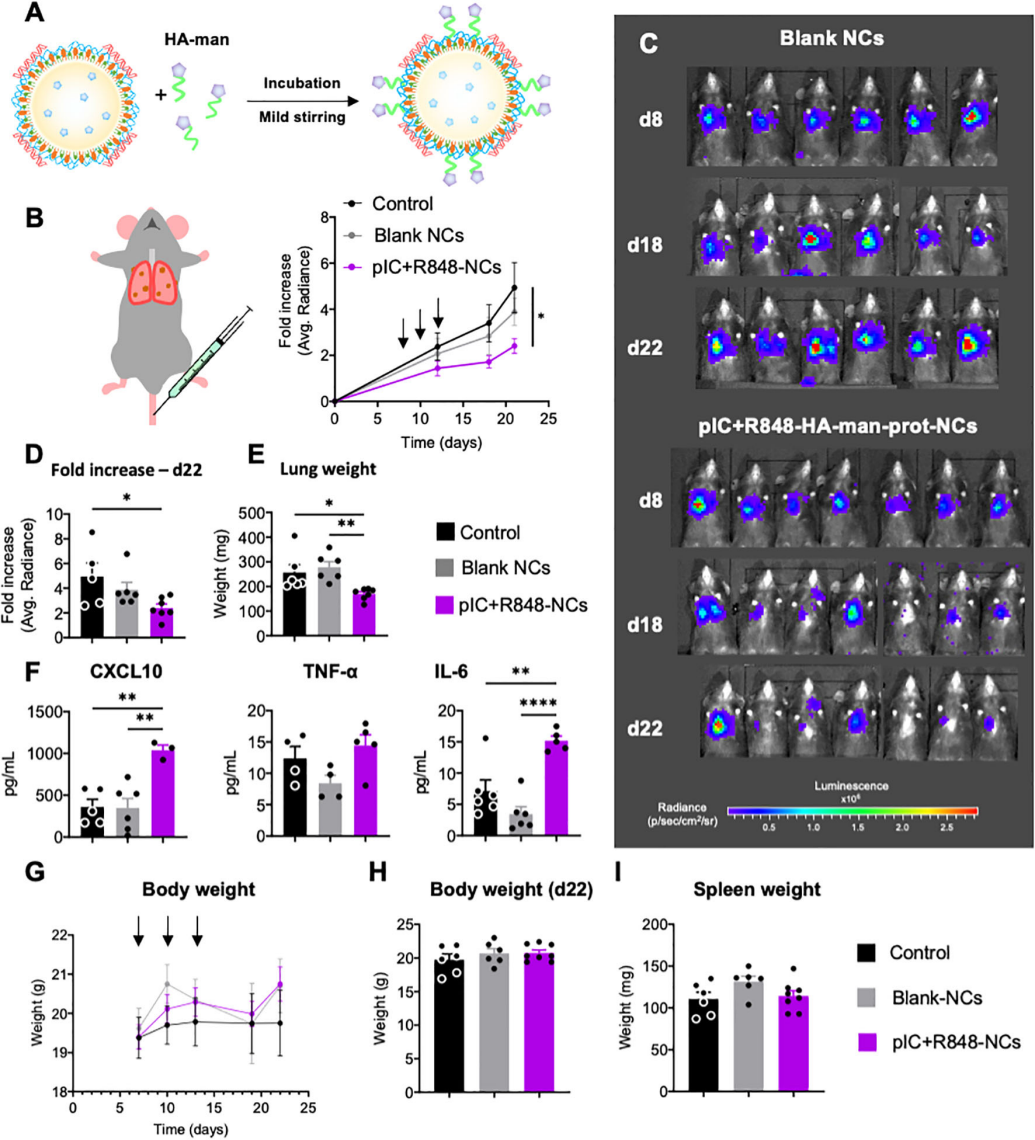
Antitumoral efficacy of poly(I:C)+R848-HA-man-prot-NCs after intravenous injection in the orthotopic lung cancer murine model CMT167. **(A)** Schematic representation of the coating of the pIC+R848-protamine-NCs with HA-mannose (here abbreviated as pIC+R848-NCs), and description of their components. **(B)** Evolution of luminescence/tumor signal of CMT167-Luc tumor-bearing mice treated with the NCs (3 intravenous injections corresponding to 25 µg of each drug at times indicated by arrows). **(C)** Representative pictures of the luminescence signal in the whole animal at 8, 18 and 22 days. **(D)** Comparison of the tumor signals in each treatment group at sacrifice. **(E)** Comparison of the lungs weights at sacrifice. **(F)** Quantification of circulation levels of CXCL10, TNF-α and IL-6 in the peripheral blood collected at day 18. **(G)** Body weight of CMT167-Luc tumor-bearing mice treated with the NCs (3 intravenous injections corresponding to 25 µg of each drug at times as indicated in Figure 5) along the whole experiment (average of 6 mice), and **(H)** last measurement at day 22. **(I)** Spleen weight at sacrifice (day 22) (each point represents 1 mouse). Values represent mean ± s.e.m. Statistical comparison was performed using a one-way ANOVA followed by a Tukey’s multiple comparison test. Statistically significant differences are represented as *(p<0.05), **(p<0.01) and ****(p<0.0001). pIC, poly(I:C); R848, resiquimod. pIC+R848-NCs, pIC+R848-protamine-NCs with HA-mannose. Blank-NCs, equivalent nanoformulations, coated with HA-mannose, but not loaded with the drugs pIC+R848.

Due to their positive surface charge, protamine-NCs would likely aggregate after intravenous (i.v.) injection. In a pilot experiment with tumor-free animals we observed difficulties to breath and physical discomfort after the injection, thus we abandoned the i.v. use of poly(I:C)+R848-prot-NCs (results not shown). To solve this issue, and to improve the ability of NCs to target TAMs in the TME of solid tumors after their i.v. injection, we took advantage of our recent work about hyaluronic acid functionalized with mannose (HA-Man) (40), and we applied an additional coating on the surface of the poly(I:C)+R848-prot-NCs with a layer of HA-Man (**Figure 5A**). For this, the HA-man was synthetized (with an 8% degree of substitution) and added in solution on the poly(I:C)+R848-prot-NCs, achieving an additional coating layer on the NCs by simple incubation (LbL: Layer-by-Layer method) (41, 56). Different mass ratios HA-man:protamine were tested to achieve an optimal coating of the NCs. Ratios from 0.5:1 to 4:1 were effective in producing a change in Z-potential from positive to negative (**Figure S8**). Final ratio of HA-man:protamine depended on the encapsulation efficiency and was kept in the same range. The particle size did not change after the addition of HA (110 nm, approximately) but Z-potential changed from positive (+12 mV) to negative (-15 mV) due to the negative charge of HA. The ability of these poly(I:C)+R848-HA-man-prot-NCs to polarize macrophages towards an M1 phenotype was also evaluated *in vitro*. HMDMs treated with poly(I:C)+R848-HA-man-prot-NCs showed similar secretion of CXCL10 and CCL5, when compared to NCs coated with HA (non-functionalized) or non-coated (**Figure S9A**). Furthermore, the ability of macrophages to kill cancer cells *in vitro* was also similar for the 3 prototypes (**Figure S9B**), guaranteeing the antitumoral activity of the poly(I:C)+R848-prot-NCs despite their coating with HA or HA-man.

For the testing of poly(I:C)+R848-HA-man-prot-NCs by intravenous injection, we selected the CMT167 orthotopic murine model of lung cancer, which is more relevant for clinical translation than subcutaneous tumor models. To follow tumor growth in the lungs CMT167-Luc cells implanted by intravenous administration were used, and they were monitored by IVIS *in vivo* imaging until sacrifice (**Figure 5B**). These measurements showed significant antitumoral activity for the poly(I:C)+R848-HA-man-prot-NCs, not observed for the blank-HA-man-prot-NCs (**Figures 5B–D**); the weight of tumor-bearing-lungs is shown in **Figure 5E**.

Along the experiment mouse behavior has been monitored, showing no apparent toxicity in the treated groups. To further evaluate potential treatment toxicity, a Luminex equipment was used to quantify cytokines in plasma collected at midtime of the experiment (day 18). A significant increase in CXCL10 was observed in mice treated with poly(I:C)+R848-HA-man-prot-NCs compared to control groups, possibly indicating the systemic activation of antitumor immune responses; similarly, the IL-6 levels were increased, but not those of TNF-α (**Figure 5F**). Total body weight did not present any significant changes along the whole experiment (**Figure 5G**), and weight of all mice and spleens were similar at the end of the experiment (**Figures 5H, I**). Furthermore, tissue sections from kidneys, liver, spleen and heart excised from one representative mouse from each treatment group showed no significant histological differences, thus validating the absence of systemic toxicity after intravenous treatments (**Figure S10**).

**Figure 6:**
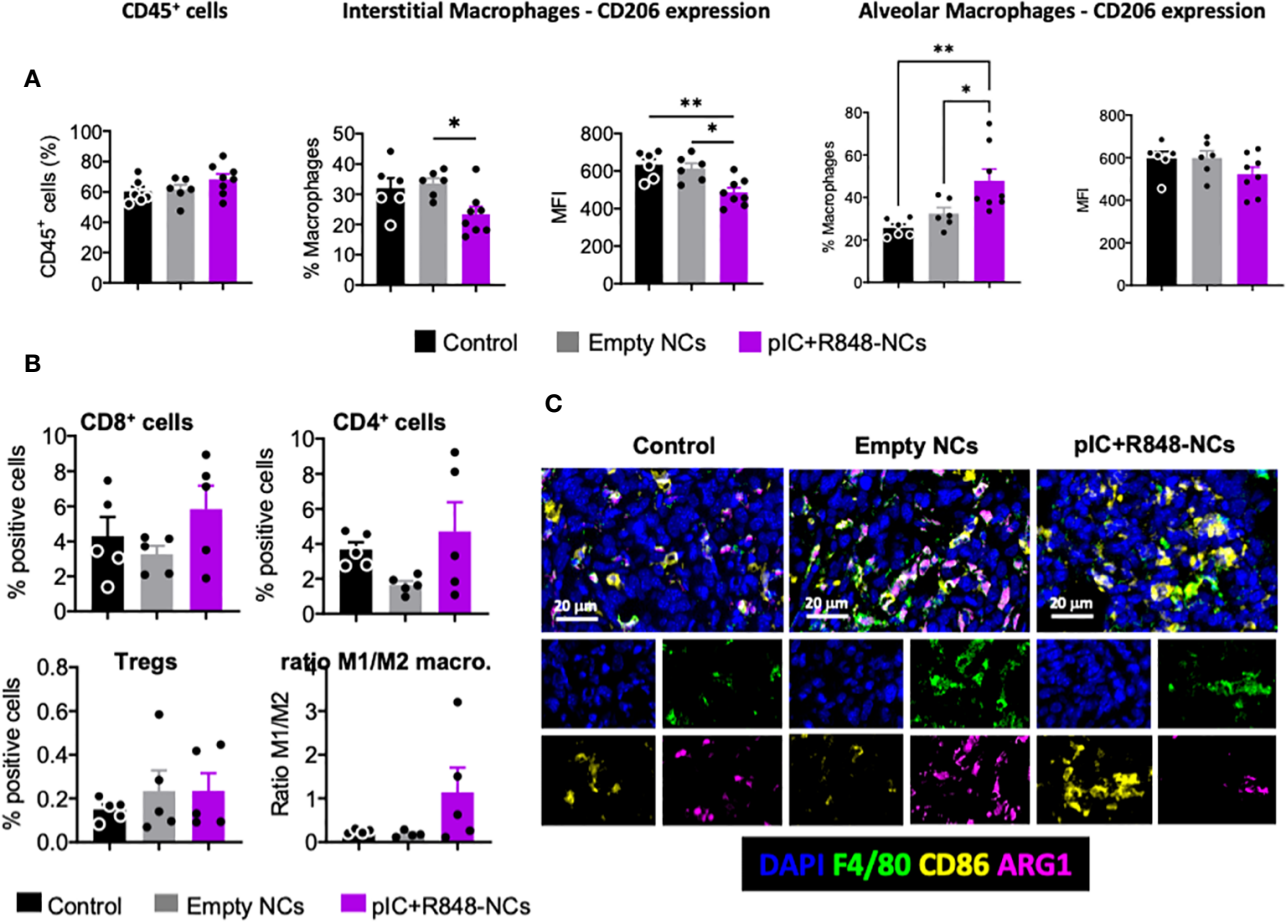
Tumor microenvironment analysis, with a focus on TAMs, after intravenous injection of poly(I:C)+R848-HA-man-prot-NCs in the orthotopic lung cancer murine model CMT167. **(A)** Flow cytometry analysis of single-cell suspensions of tumor-bearing lungs: total leukocytes (CD45^+^) among living cells, and quantification of interstitial macrophages (CD64^+^; Cd11b^high^) among total macrophages, and CD206 expression; quantification of alveolar macrophages (CD64^+^; Cd11b^low^) among total macrophages, and CD206 expression. **(B, C)** Multiplexed immunofluorescence analysis of CMT167-Luc-derived tumors at sacrifice. Quantification of CD8^+^ cells, CD4^+^ cells, FOXP3^+^/CD4^+^ Treg cells and ratio of M1 (F4/80^+^, CD86^+^):M2 (F4/80^+^, Arg1^+^) macrophages in treated tumors. **(C)** Representative images of controls, tumors treated with blank-NCs or with pIC+R848-NCs. DAPI was used for staining of nucleus, F4/80 for macrophages, CD86 as M1 marker and Arg1 as M2 marker. Values represent mean ± s.e.m. Statistical comparison was performed using a one-way ANOVA followed by a Tukey’s multiple comparison test. Statistically significant differences are represented as *(p<0.05) and **(p<0.01). pIC, poly(I:C); R848, resiquimod. pIC+R848-NCs, pIC+R848-protamine-NCs with HA-mannose. Blank-NCs, equivalent nanoformulations, coated with HA-mannose, but not loaded with the drugs pIC+R848.

To evaluate the immune infiltration in the lung-TME and TAM-reprogramming upon treatment, FACS and multiplex imaging analysis were performed on excised lungs. The analysis of the TME by flow cytometry showed no significant differences in the total population of immune cells (CD45+) (**Figure 6A**), neither a significant change in the percentage of macrophages (F4/80+) in poly(I:C)+R848-HA-man-prot-NCs treated mice (results not shown). However, TAMs from mice treated with poly(I:C)+R848-HA-man-prot-NCs had a significant decrease in the interstitial-monocyte-derived/recruited macrophages (CD64^+^; Cd11b^high^), *versus* the alveolar/resident macrophages (CD64^+^; Cd11b^low^) (**Figure 6A**). More interestingly these recruited macrophages presented a significantly lower expression of CD206 (M2 marker) in response to the treatment while no significant differences were observed for CD206 in the alveolar macrophages (**Figure 6A**). Multiplex immunofluorescence analysis of the TME showed no significant changes in the infiltration of T cells (neither CD4, CD8 or Treg populations were considerably affected) (**Figure 6B**), although in mice treated with poly(I:C)+R848-HA-man-prot-NCs a high variability in the number of CD4 and CD8 T cells was observed within the same group. Importantly, the multiplex immunofluorescence methodology clearly confirmed, in treated mice, the reprogramming of macrophages towards an M1-like antitumor phenotype, by the increase in the ratio of M1 (F4/80^+^, CD86^+^):M2 (F4/80^+^, Arg1^+^) macrophages (**Figures 6B, C**). As a whole, the intravenous injection of poly(I:C)+R848-HA-man-prot-NCs showed high antitumoral activity, mediated by TAM reprogramming in the TME, in a pre-clinical orthotopic murine model of lung cancer without any systemic toxicity.

## Discussion

In this study, we have developed and evaluated the antitumoral and immunomodulatory activity of polymeric NCs loaded with TLR agonists to reprogram TAMs in solid tumors. TLR agonists have been extensively investigated for their capacity to generate antitumoral responses, mainly in the field of cancer vaccination, and several pharmaceutical formulations are currently in clinical trials (13, 16). However, the activity of these therapies on TAMs has been hardly explored (5, 14). Other approaches, such as monoclonal antibodies or small drugs are in clinical trials to target TAMs (3–6, 10–12). Our group previously reported that the poly(I:C)+R848 combination, *versus* monotherapy or other TLR agonist combinations, presents a superior ability to reprogram and to induce antitumoral activity *in vivo* (29). Therefore, here we have developed and evaluate *in vitro* and *in vivo* new nanosystems allowing the encapsulation of both drugs in similar amounts (ratio 1:1). Comprehensive characterization of the initial NCs developed using 5 different types of polymers showed similar properties in terms of particle size, but great differences in surface charge (from -38 till +55 mV), and encapsulation efficiencies for the R848 around 15%. The *in vitro* experiments with primary human macrophages led to the selection of the protamine-NCs as the optimal formulation characterized by its size (|100 nm), more neutral surface charge (+ 18 ± 9 mV) and R848-loading (12 ± 4%) presenting the excellent immunostimulatory properties and the best biocompatible profile. The positive charge of these NCs allowed the easy loading of poly(I:C) (negatively charged dsRNA), and also their additional coating with a protective layer of HA or HA-mannose which adds functional targeting properties to target the mannose receptor (CD206) overexpressed on the surface of TAMs in the TME, as recently reported (29).

To our knowledge, the loading of both drugs (poly(I:C)+R848) into an unique nanocarrier has only been attempted before by Da Silva et al, who co-encapsulated both TLR agonists with antigens, chemokines or cytotoxic drugs in PLGA-derived-nanoparticles (57). However, in their studies the activity on TAMs was not evaluated. Our experiments *in vitro* confirmed the ability of poly(I:C)+R848-prot-NCs, with or without HA-mannose coating, to stimulate macrophages towards an M1-like antitumoral mode, characterized by secretion of CXCL10 and CCL5 and also increased cytotoxic activity towards cancer cells. For the *in vivo* experiments, we decided to follow recent work by our group and others on intratumoral administration of TLR agonists (14, 58). Importantly, local (i.t.) delivery of TLR agonists showed powerful effects not only against the injected tumors but also often against uninjected lesions (abscopal effects) and distant metastasis. Using this approach, we demonstrated the antitumoral efficacy of poly(I:C)+R848-prot-NCs in two immunocompetent murine tumor models, with a higher reduction in the growth of the primary tumor in the more immunogenic subcutaneous lung cancer model (CMT167) *versus* the more resistant and fast-growing orthotopic fibrosarcoma model (MN/MCA). Of note, systemic antitumor immunity was found also in both models, evaluated by activation of splenocytes in the CMT167 model and prevention of lung metastasis in the MN/MCA tumors. Previous studies have reported the increase of metastasis by TLR3 or TLR7 activation (59), while metastasis prevention was observed by treatment with numerous TLR agonists often in combination with chemotherapy or radiotherapy (16). Interestingly, McCormick et al. found that activation of TLR3 prior to metastasis inhibited migration of cancer cells, while its activation during metastasis enhanced their migration (60). Based on our results, we could hypothesize that intratumoral administration of poly(I:C)+R848-protamine-NCs unequivocally activate the antitumor immune abscopal effect, which is not obvious in the case of the same NCs loaded only with poly(I:C). Further studies, using other metastatic tumor models, other posology, and routes of administration, with further characterization of systemic activity are needed.

Finally, the orthotopic lung cancer model CMT167 was used to evaluate the antitumoral efficacy of poly(I:C)+R848-HA-man-prot-NCs, designed for intravenous (i.v.) administration to target TAMs in the TME. Recent work by others has been mainly focused on the development of nanosystems for the i.v. delivery of R848 towards TAMs (18–20), but not poly(I:C), which has been mainly applied in the form of nanocomplexes through i.t. administration (23–26). Our results show significant reduction of lung tumors and activation of systemic immunity (CXCL10 and IL-6, but not TNF-α), with no apparent toxicity. Furthermore, the characterization of the TME confirmed the key role of TAM reprogramming, while no significant changes were observed in the number of T cells. Most *in vivo* studies with TLR agonists or other immunotherapies, have reported cytotoxic CD8 T cells as major contributors to the antitumoral efficacy (13, 14), while CD4 T cells might be more important for immune memory and sustained antitumoral responses (61, 62). However, the numbers and phenotype of T cells might vary along the course of treatment, thus we consider that more exhaustive analysis at different times are needed to understand the involvement of the adaptive immune system in the antitumor activity. In this orthotopic model of lung cancer, we have injected the CMT167 cells through an i.v. tail injection. Once the cells are seeded in the lungs, they might form other tumors along the whole lung tissue, encountering alveolar/resident macrophages which are polarized towards an M2-immunosuppressive phenotype by the tumor cells. Furthermore, these solid tumors trigger the recruitment of new interstitial-monocyte-derived macrophages, mainly generated in the bone marrow, which also present an immunosuppressive phenotype with high expression of typical M2-markers, such as CD206. Interestingly, in addition to the increase in the M1 (F4/80^+^, CD86^+^):M2 (F4/80^+^, Arg1^+^) ratio observed in the TME, we have also been able to differentiate the higher activity of the poly(I:C)+R848-HA-man-prot-NCs on the newly recruited interstitial-monocyte-derived macrophages quantified as decreased number of these cells in the lung tumors and also lower expression of CD206. This response to the treatment can be considered beneficial for the prevention of metastasis because interstitial-recruited macrophages from monocyte origin presenting an immunosuppressive phenotype driven by the tumor (Cd11b^high^), have been associated with enhanced tumor spreading (63). Further experiments using more clinically relevant metastatic tumor models could be helpful to understand with more detail the mechanism of action of our therapy, but also the dynamics of TAMs in lung tumors, and this exhaustive work remains out of the scope of this manuscript.

In conclusion, our study highlights the importance of performing side-by-side formulation development and *in vitro* evaluation, as parallel tasks providing useful feedback that allows the optimization of the drug delivery nanosystems for a particular biological activity. Our polymeric NCs, presented a similar size (around 100 nm) and ability to encapsulate R848 in their oily core (10-20% EE), but a huge difference in their zeta-potential (from -38 till +55 mV). While the nature of the polymer, on their surface, and the physicochemical properties of the R848-loaded-NCs did not significantly influence their *in vitro* toxicity profile; instead, their ability to polarize HMDMs towards an M1-like antitumoral phenotype was quite variable showing the best results for the protamine-NCs (in terms of secretion of pro-inflammatory cytokines CXCL10, CCL5 and IL-6). Addition of poly(I:C) and NC-coating with a layer of HA functionalized with mannose to target TAMs, was implemented taking advantage of their electrostatic interactions with the protamine by simple incubation and mild stirring, resulting in a NC-formulation with optimal properties and high stability for their *in vivo* administration. Using two different murine tumor models, our experiments demonstrated that the i.t. administration of poly(I:C)+R848-prot-NCs showed potent antitumor efficacy and ability to prevent spontaneous lung metastasis with evidence of systemic activation of antitumor immunity; the intravenous administration of poly(I:C)+R848-HA-man-prot-NCs reduced the growth of orthotopic lung tumors through demonstrated reprogramming of interstitial macrophages increasing the M1/M2 ratio. As a whole, our new polymeric NCs for the *in vivo* administration of TLR agonists poly(I:C)+R848, trigger local and systemic antitumor immunity mediated by TAM reprogramming for the treatment of solid tumors.

## Data availability statement

The original contributions presented in the study are included in the article/**Supplementary Material**. Further inquiries can be directed to the corresponding author.

## Ethics statement

The studies involving humans were approved by IRCCS Humanitas Research Hospital ethics committee. The studies were conducted in accordance with the local legislation and institutional requirements. The ethics committee/institutional review board waived the requirement of written informed consent for participation from the participants or the participants’ legal guardians/next of kin because All samples were anonymous and destroyed at the end of the experiments. No data from human donors are available in our manuscript. The animal study was approved by IRCCS Humanitas Research Hospital ethics committee, autorization 453/2020-PR and 909/2023-PR. The study was conducted in accordance with the local legislation and institutional requirements.

## Author contributions

CA: Formal analysis, Investigation, Methodology, Visualization, Writing – original draft, Writing – review & editing. CFV: Methodology, Writing – original draft. AU: Methodology, Writing – original draft. AM: Investigation, Methodology, Writing – original draft. MS: Investigation, Methodology, Writing – original draft. SG: Investigation, Methodology, Writing – original draft. JB: Investigation, Methodology, Writing – original draft. ML: Investigation, Methodology, Writing – original draft. SL: Investigation, Methodology, Writing – original draft. AC: Investigation, Methodology, Resources, Writing – original draft, Writing – review & editing. JC: Investigation, Methodology, Writing – original draft. EF-M: Investigation, Methodology, Resources, Writing – original draft. MA: Investigation, Resources, Writing – review & editing. PA: Resources, Writing – review & editing. JC-C: Investigation, Methodology, Writing – original draft, Writing – review & editing. FA: Conceptualization, Formal Analysis, Funding acquisition, Investigation, Methodology, Project administration, Resources, Supervision, Visualization, Writing – original draft, Writing – review & editing.
