# Supplementary material for: Polymeric nanocapsules loaded with poly(I:C) and resiquimod to reprogram tumor-associated macrophages for the treatment of solid tumors
Source: Front Immunol. 2024 Jan 8;14:1334800. doi: 10.3389/fimmu.2023.1334800 (PMC10800412; doi:10.3389/fimmu.2023.1334800)
Supplement: Supplementary file 1 [file DataSheet_1.pdf]

Supplementary Material

Polymeric nanocapsules loaded with poly(I:C) and resiquimod to reprogram tumor-associated macrophages for the treatment of solid tumors

Clément Anfray[1], Carmen Fernández Varela[2], Aldo Ummarino[1], Akihiro Maeda[1], Marina Sironi[1], Sara Gandoy[2-3], Jose Brea[3], María Isabel Loza[3], Sergio León[4], Alfonso Calvo[4], Juan Correa[5], Eduardo Fernandez-Megia[5], María José Alonso[2†], Paola Allavena[1†], José Crecente-Campo[2†], Fernando Torres Andón[1,2,6†]

[1]Laboratory of Cellular Immunology, IRCCS Humanitas Research Hospital, Rozzano-Milan 20089, Italy
[2]Center for Research in Molecular Medicine and Chronic Diseases (CiMUS), Campus Vida, Universidade de Santiago de Compostela, Santiago de Compostela 15782, Spain
[3]BioFarma Research Group, CIMUS, Departamento de Farmacología, Farmacia y Tecnología Farmacéutica. Facultad de Farmacia. Universidade de Santiago de Compostela, Santiago de Compostela 15782, Spain
[4]IDISNA, Program in Solid Tumors, Center for Applied Medical Research (CIMA), Department of Pathology and Histology, University of Navarra, 31008 Pamplona, Spain; CIBERONC, Madrid, Spain
[5]Centro Singular de Investigación en Química Biolóxica e Materiais Moleculares (CIQUS), Departamento de Química Orgánica, Universidade de Santiago de Compostela, Jenaro de la Fuente s/n, 15782 Santiago de Compostela, Spain
[6]Instituto de Investigación Biomédica de A Coruña (INIBIC), Oncology Department, Complexo Hospitalario de A Coruña (CHUAC), 15006 A Coruña, Spain.

[†]María José Alonso, Paola Allavena, José Crecente Campo and Fernando Torres Andón contributed equally to this work and share senior authorship.

*** Correspondence:**
Fernando Torres Andón, PhD
Instituto de Investigación Biomédica de A Coruña (INIBIC), Oncology Department, Complexo Hospitalario de A Coruña (CHUAC), As Xubias s/n, 15006, A Coruña, Spain.
E-mail: fernando.torres.andon@sergas.es ORCID: 0000-0001-9235-1278

This is a provisional file, not the final typeset article

Compound	Final concentration (mg/mL)	
	**Blank**	**R848-Loaded**
DL-α-Tocopherol	6.75	6.75
TPGS	2	2
Sodium cholate or<br>*Benzethonium chloride*	0.5 or<br>0.25	0.5 or<br>0.25
R848	-	1
Polymer	1	1

Table S1. Composition of polymeric nanocapsules (NCs) before isolation and concentration.

This is a provisional file, not the final typeset article

IC_{50} (µg/ml)	<100	100 – 1000	>1000
Color code			

	M0		M1		M2	
**Polymeric nanocapsules**	1h + 24h	24h	1h + 24h	24h	1h + 24h	24h
Poly-Arginine NCs	572,1	302,8	573,4	226,9	328,8	150,2
Chitosan NCs	> 1000	856,8	> 1000	827,1	> 1000	> 1000
Protamine NCs	> 1000	> 1000	> 1000	> 1000	> 1000	> 1000
Dextran Sulfate NCs	> 1000	110,9	> 1000	46,3	> 1000	61,1
Poly-Sialic Acid NCs	342,2	83,8	138,1	100,4	646,1	130,5

Table S2. IC50 of blank polymeric nanocapsules in M0, M1 and M2 polarized primary human monocyte derived macrophages (HMDMs). Cells were prepared as described in methodology. After polarization, macrophages were exposed to the NCs for 1h, washed and then cultivated for 24 hours in cell culture medium (RPMI + 10% FBS + 1xPSG) at 37ºC (left column, 1h + 24 h) or directly exposed to the cells for 24 hours under the same conditions, without washing (right column, 24h). Concentrations of total NC-components were used as follows: 0, 0.1, 1, 10, 100 and 1000 μg/ml. Toxicity for the calculation of IC50 (half maximal inhibitory concentration) was measured by Alamar Blue assay with the equation: cell viability (%) = (1 – fluorescence/control fluorescence) * 100.

	M0		M1		M2	
**Polymeric nanocapsules**	1h + 24h	24h	1h + 24h	24h	1h + 24h	24h
Poly-Arginine NCs	469,3	539,9	640,2	752,4	535,7	757,1
Chitosan NCs	> 1000	> 1000	> 1000	> 1000	> 1000	> 1000
Protamine NCs	> 1000	> 1000	> 1000	> 1000	> 1000	> 1000
Dextran Sulfate NCs	> 1000	> 1000	> 1000	798,1	> 1000	> 1000
Poly-Sialic Acid NCs	> 1000	> 1000	> 1000	754,2	> 1000	> 1000

Table S3. IC50 of blank polymeric nanocapsules in M0, M1 and M2 polarized THP-1 cells. Cells were prepared as described in methodology (cell culture models), and treated as described in S2. Same doses and times of exposure were used, and also Alamar Blue assay was used to evaluate toxicity and to calculate the IC50.

	R848 concentration (µg/ml)			
	**0.5**	**1**	**5**	**10**
R848-Poly-Arg-NCs	32	64	320	640
R848-Chit-NCs	28	56	280	560
R848-Prot-NCs	28	56	280	560
R848-Dext-NCs	26	52	260	520
R848-PSA-NCs	26	52	260	520

Table S4. Correspondence between R848 concentration and concentration of total nanocapsule concentration (considering all the components) in R848-loaded polymeric nanocapsules (µg/ml).

This is a provisional file, not the final typeset article

Antibodies	Reference	Dilution
CD45-PerCP	Clone 30-F11 (BD Biosciences)	1:200
Cd11b-APC eFluor780	Clone M1/70 (eBiosciences)	1:400
F4/80-PE	Clone CI:A3-1 (BioRad)	1:50
CD64-PEcy7	Clone X54-5/7.1 (BioLegend)	1:250
CD206-BV421	Clone C068C2 (BioLegend)	1:500

Table S5. List of antibodies used for flow cytometry in experiments presented in Figure 6A.

Antibody (Reference)	*Dilution*	*Antigen retrieval*	*Opal / Dilution*
FOXP3 (CST, 12653)	1:600	Citrate Buffer, pH6	480 / 1:100
CD4 (Abcam, ab183685)	1:400	Citrate Buffer, pH6	520 / 1:300
CD8 (CST, 98941)	1:500	Citrate Buffer, pH6	620 / 1:100
F4/80 (CST, 70076)	1:400	Citrate Buffer, pH6	570 / 1:600
CD86 (CST, 19589)	1:300	Citrate Buffer, pH6	690 / 1:100
Arg1 (CST, 936668)	1:200	Tris-EDTA Buffer, pH9	620 / 1:100

Table S6. Antibodies and conditions used for multispectral immunophenotyping in experiments presented in Figure 6B and 6C.

A)

Blank polymeric nanocapsules	Particle size (nm)	PDI	Z-potential (mV)	pH
Poly-Arginine NCs	146 ± 8	0.10	+ 51 ± 2	6
Chitosan NCs	150 ± 7	0.11	+ 16 ± 6	5
Protamine NCs	140 ± 6	0.11	+ 5 ± 8	6
Dextran Sulfate NCs	142 ± 4	0.11	- 15 ± 2	7
Poly-Sialic Acid NCs	151 ± 11	0.13	- 42 ± 5	6

B)

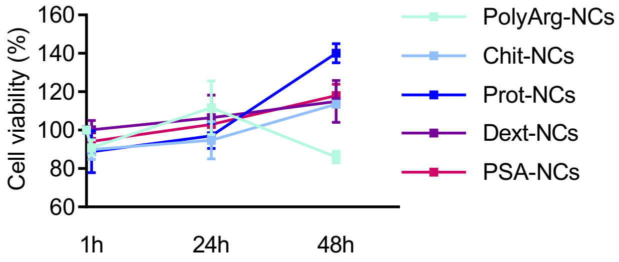

C)

Polymeric NCs	IC_{50} (µg/mL)
PolyArg-NCs	302.8
Chitosan-NCs	856.8
Protamine-NCs	>1000
Dextran Sulfate-NCs	110.9
Poly-Syalic Acid-NCs	83.8

Figure S1. Blank polymeric nanocapsules (NCs): characterization and in vitro toxicological evaluation. (A) Physicochemical characteristics of blank nanocapsules prepared with different outer polymers (n ≥ 3). **(B)** Toxicological kinetics of blank polymeric NCs (10 µg/ml) towards HMDMs; N=3. **(C)** IC50 values of blank-NCs in HMDMs (24h); N=4. All values represent mean ± SD. NCs, nanocapsules; PolyArg, polyarginine; Chit, chitosan; Prot, protamine; Dex, dextran sulfate; PSA, poly-sialic-acid.

This is a provisional file, not the final typeset article

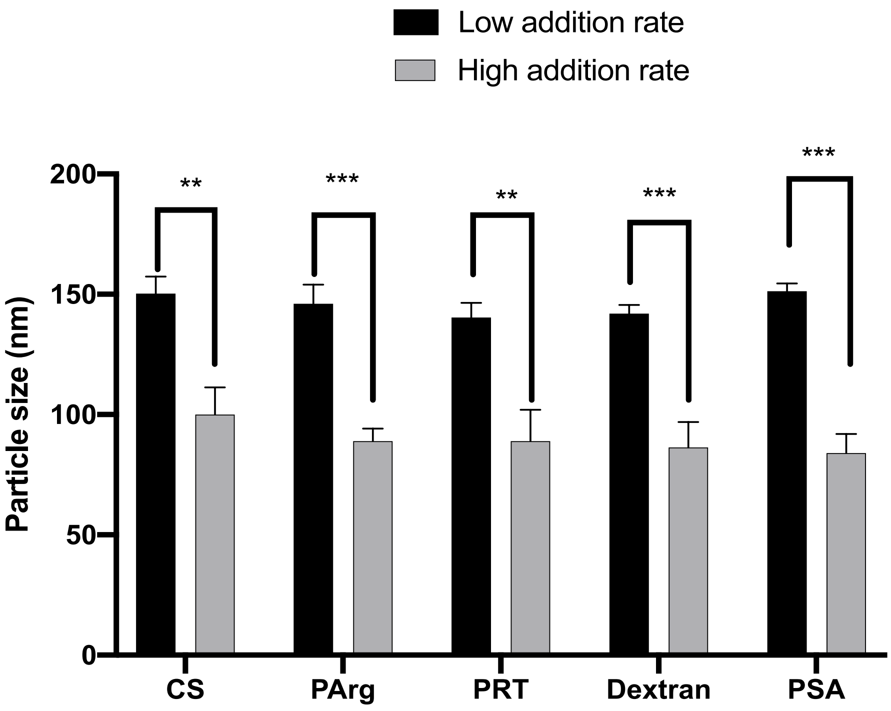

Figure S2. Optimization of particle size of blank nanocapsules prepared with different polymers. Statistical significance determined using the Holm Sidak method, with alpha = 0.05. Each row was analyzed individually, without assuming a consistent SD. Number of t tests: 5. ** p = <0,01, *** p = <0,001, **** p = <0,0001. CS: Chitosan, PArg: Poly-Arginine, PRT: Protamine, Dex: Dextran Sulfate, PSA: Poly-Sialic Acid.

This is a provisional file, not the final typeset article

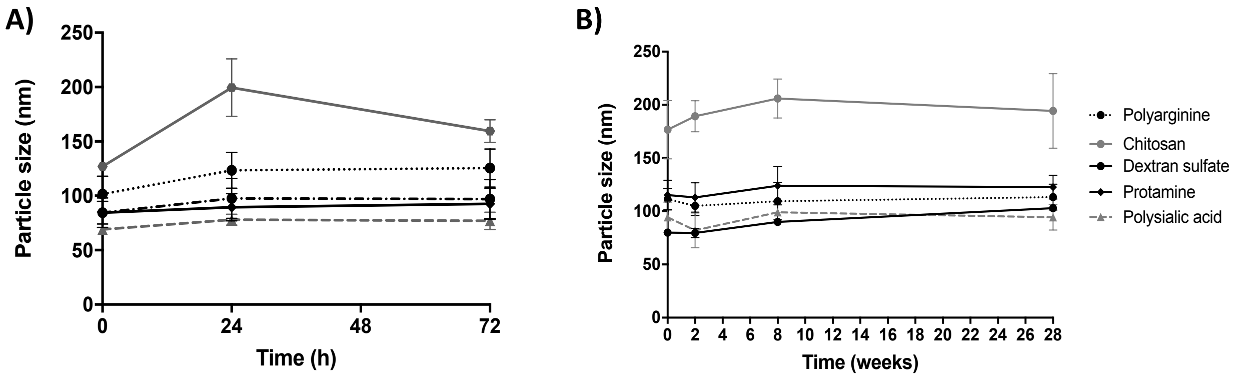

Figure S3. Stability of blank polymeric nanocapsules, measured as determination of particle size by DLS, in A) cell culture media (RPMI + 10% FBS + 1xPSG) at 37ºC or B) in storage conditions at 4ºC (n=3).

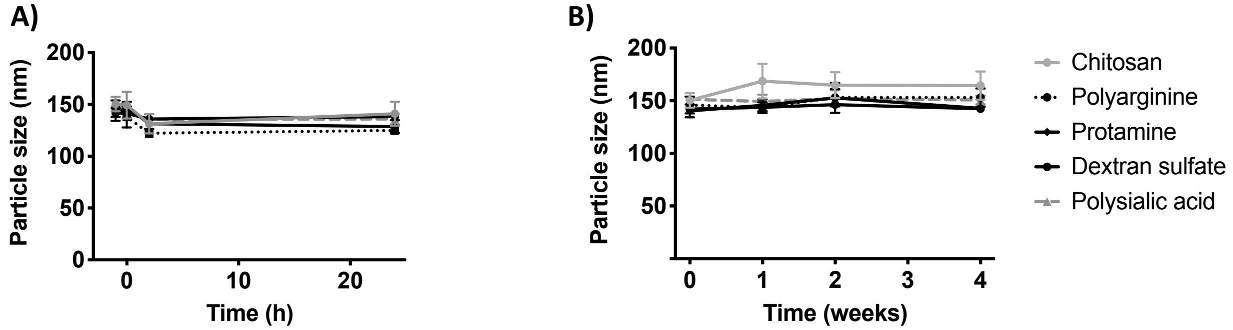

Figure S4. Stability of R848-loaded polymeric nanocapsules, measured as determination of particle size by DLS, in A) cell culture media (RPMI + 10% FBS + 1xPSG) at 37ºC or B) in storage conditions at 4ºC (n=3).

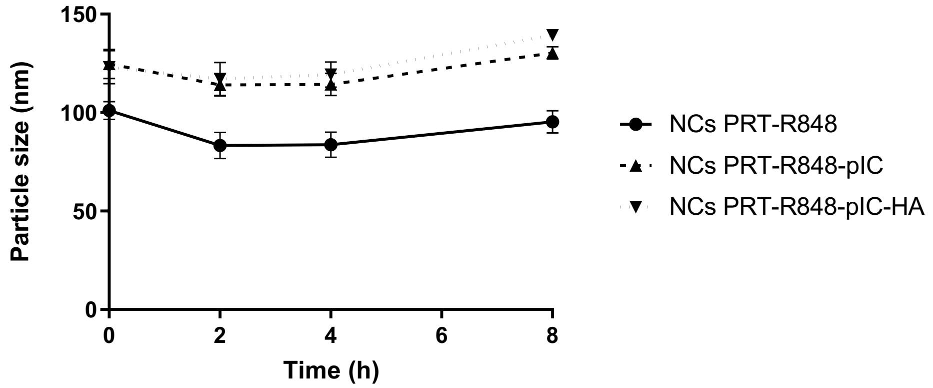

Figure S5. Stability of R848-protamine-NCs, poly(I:C)+R848-protamine-NCs and HA-coated poly(I:C)+R848-protamine-NCs, determined as particle size measured by DLS, in simulated physiological fluids (PBS 10%FBS) (n=3). NCs, nanocapsules; PRT, protamine; pIC, poly(I:C); R848, resiquimod; HA, hyaluronic acid).

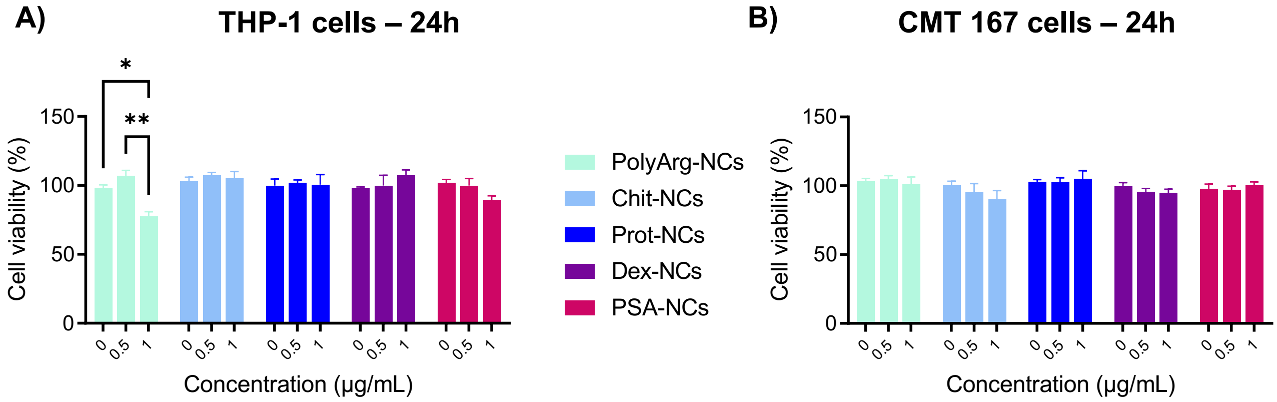

Figure S6. Toxicological evaluation of polymeric NCs loaded with R848 using THP-1 and CMT167 cell lines evaluated by Alamar Blue assay. (A) Toxicity on THP-1 macrophages exposed 24h to the R848-loaded-NCs. **(B)** Toxicity on CMT167 cells exposed 24h to the R848-loaded-NCs. Values represent mean ± s.e.m, N=6. Statistical comparison was performed using a two-way ANOVA followed by a Dunnett's multiple comparison test. Statistically significant differences are represented as *($p<0.05$) and **($p<0.01$) vs 0 µg/ml. R848: Resiquimod, NCs: nanocapsules, PolyArg: Poly-Arginine, Chit: Chitosan; Prot: Protamine, Dex: Dextran Sulfate, PSA: Poly-Sialic Acid.

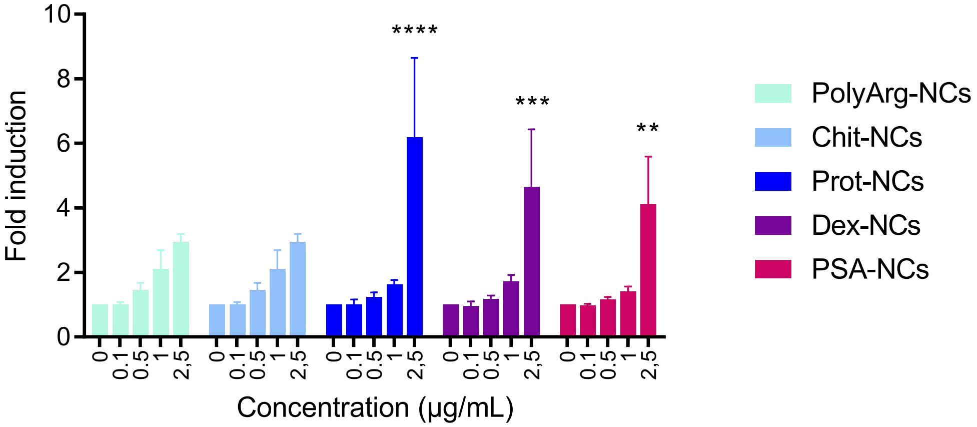

Figure S7. Activation of the NF-kB pathway in THP-1 cells exposed for 24 hours to R848 loaded polymeric NCs. THP1-Lucia cells QUANTI-LucTM, luciferase detection reagent, were acquired from InvivoGen. The reporter THP1-Lucia cells have been specifically designed for monitoring the activation of the NF-κB signal transduction pathway in a physiologically relevant monocytic cell line by quantification of secreted luciferase (Lucia). THP1 cells were cultivated in RPMI 1640, 2 mM L-glutamine, 25 mM HEPES, 10% heat-inactivated fetal bovine serum, 100 µg/mL Normocin, 200 µg/mL Zeocin, and Pen-Strep (100 U/mL–100 µg/mL). The cells were treated with R848-loaded-NCs at indicated concentrations for 24 hours, and following manufacturer's protocols, NF-κB activation was quantified by determining Lucia luminescence and represented as relative luminescence units (RLUs). Fold induction was calculated with the following equation:

$$\text{fold induction} = (\text{RLU}/\text{RLU control}) \times 100$$

Values represent mean ± s.e.m, N=4. Statistical comparison was performed using a one-way ANOVA followed by a Tukey's multiple comparison test. Statistically significant differences are represented as *(p<0.05) and **(p<0.001). R848, resiquimod; NCs, nanocapsules; PolyArg, polyarginine; Chit, chitosan; Prot, protamine; Dex, dextran sulfate; PSA, poly-sialic-acid.

This is a provisional file, not the final typeset article

A)

HA (mg)	PRT (mg)	HA (mg/ml)	PRT (mg/ml)	Mass ratio	-/+ ratio
0.183 mg	0.367 mg	4.57 mg/ml	2.3 mg/ml	0.5:1	0.4:1
0.367 mg		9.2 mg/ml		1:1	0.7:1
0.736 mg		18.4 mg/ml		2:1	0.9:1
1.472 mg		36.8 mg/ml		4:1	2.6:1

B)

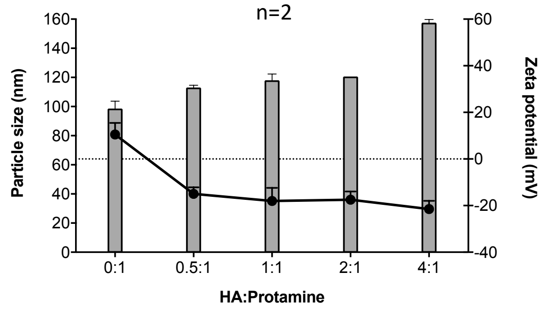

C)

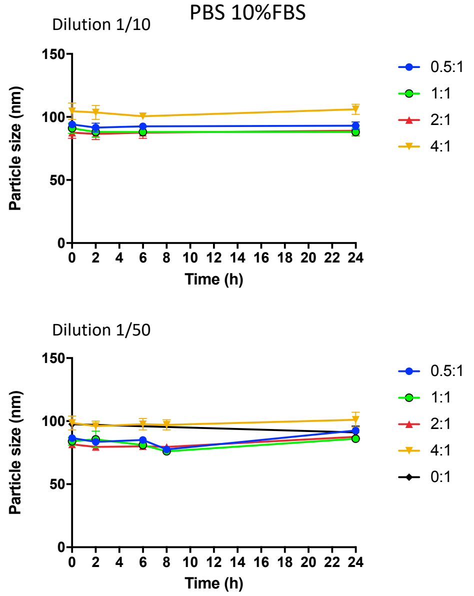

Figure S8. Development and characterization of protamine nanocapsules coated with hyaluronic acid. **(A)** Table with the amount (mg), concentration (mg/mL) and mass ratio of polymers (PRT or HA) used for development of each type of NCs. **(B)** Characterization of these prototypes as particle size (nm, y-axis left) and surface charge (mV, y-axis right). **(C)** Stability evaluation of these prototypes evaluated by characterization of their particle size after incubation up to 24 hours in PBS with 10% of FBS at 1/10 dilution (graph above) and 1/50 (graph below). NCs, nanocapsules; PRT, protamine; HA, hyaluronic acid.

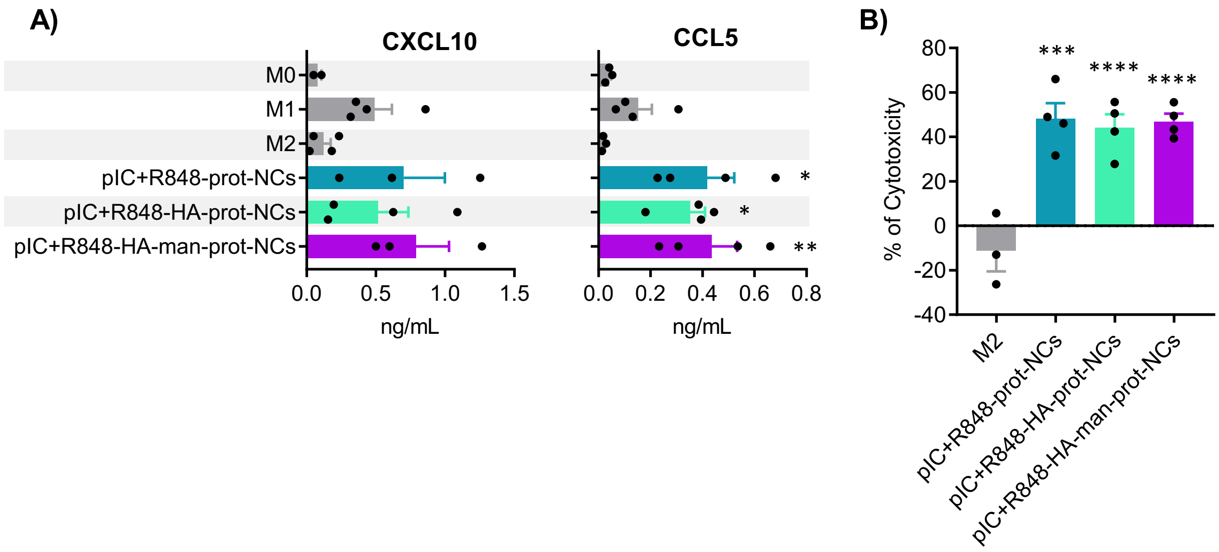

Figure S9. Functional activity of pIC+R848-HA-man-prot-NCs. (A) Secretion of CXCL10 and CCL5 and **(B)** cytotoxic activity of HMDMs towards PANC-1 cells exposed 24h to pIC+R848-prot-NCs (0.5 µg/mL), with or without functionalization by HA or HA-Mannose. Values represent mean ± s.e.m. Statistical comparison was performed using a two-way ANOVA followed by a Dunnett's multiple comparison test. Statistically significant differences are represented as *($p<0.05$), **($p<0.01$), ***($p<0.001$) and ****($p<0.0001$). NCs, nanocapsules; prot, protamine; pIC, poly(I:C); R848, resiquimod; HA, hyaluronic acid; Man, mannose.

This is a provisional file, not the final typeset article

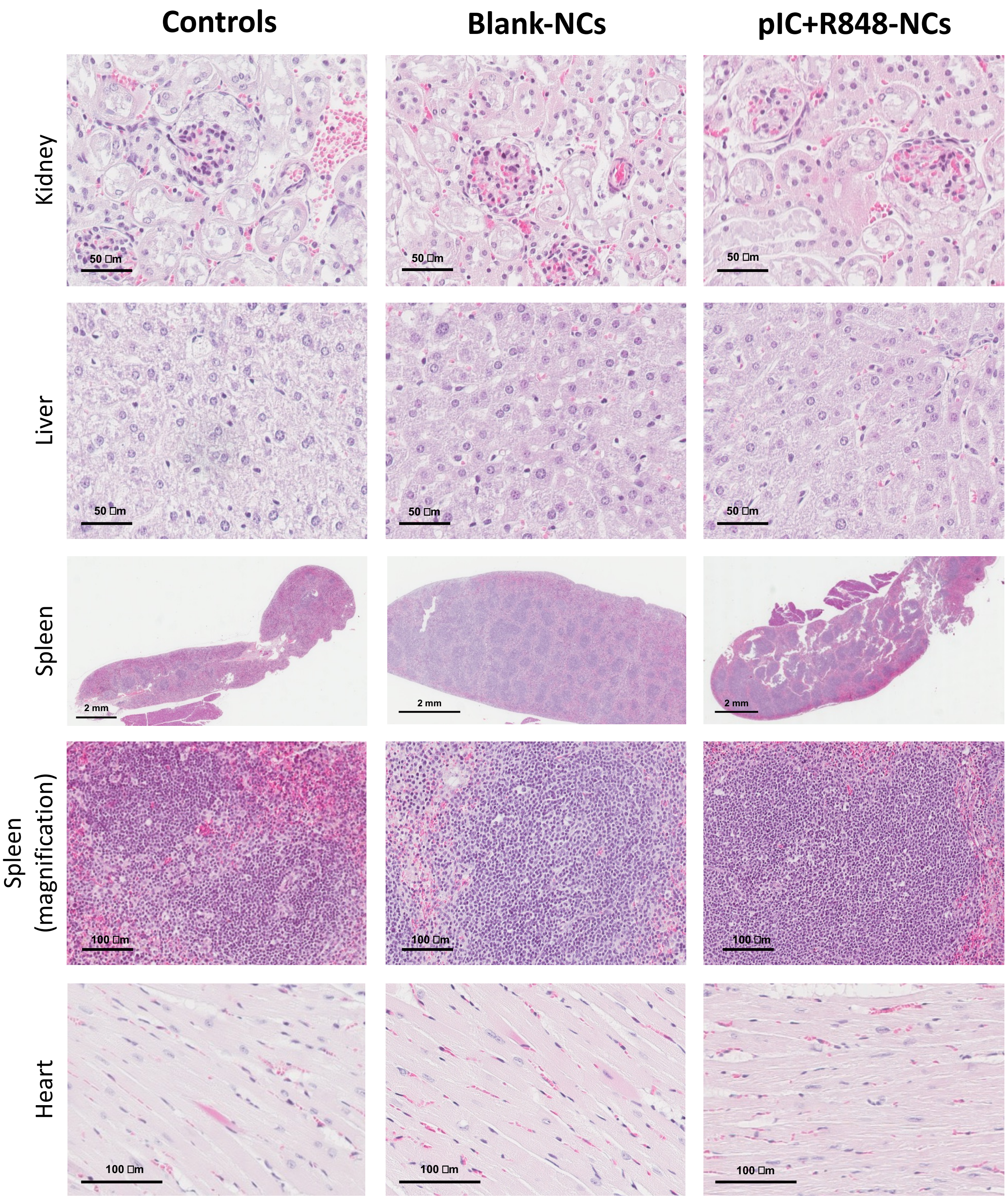

Figure S10. Immunohistological analysis by hematoxylin and eosin staining of relevant organs (kidney/liver/spleen/heart) for toxicological evaluation *in vivo* of pIC+R848-HA-Man-prot-NCs after their intravenous administration. CMT167-Luc tumor-bearing mice treated with the NCs (3 intravenous injections corresponding to 25 µg of each drug at times as indicated in Figure 5) along the whole experiment (one representative sample from each group is shown). Blank-NCs, protamine-nanocapsules coated with HA-mannose; pIC+R848-HA-man-prot-NCs, protamine-nanocapsules loaded with poly(I:C)+R848 and coated with HA-mannose.
